# Measuring evidence-based practice in physical therapy: a mix-methods study

**DOI:** 10.7717/peerj.12666

**Published:** 2022-01-04

**Authors:** Ricardo M. Ferreira, Pedro N. Martins, Nuno Pimenta, Rui S. Gonçalves

**Affiliations:** 1 Physical Exercise and Sports Department, Polytechnic Institute of Maia, Maia, Porto, Portugal; 2 Physical Therapy Department, Coimbra Health School, Polytechnic Institute of Coimbra, São Martinho do Bispo, Coimbra, Portugal; 3 Physical Exercise and Sports Department, Polytechnic Institute of Bragança, Bragança, Portugal

**Keywords:** Evidence-based practice, Physical therapy, Mix-methods

## Abstract

**Background:**

Evidence-based practice (EBP) is considered the “holy grail” to manage patients by health practitioners (such as physical therapists). However, sometimes, patients are not treated with the best interventions for their condition. Although studies already explored the facilitators and barriers for this issue, they increase in the level of importance if the information gathered are context appropriated. As the profession is relatively new in Portugal, currently little is known about the implementation of EBP in Portuguese physical therapists context. So, the aim of this study is to know if the Portuguese physical therapists use an EBP, and collect and deeper understand the factors, barriers and facilitators associated with EBP.

**Methods:**

This study incorporated a mixed-methods design (quantitative and qualitative). In an attempt to ensure the correct population sample, a national professional association e-mail database and the e-mails of past students from national schools were requested. For the quantitative data it was choose an e-survey, adapted from the EBP: Beliefs, Attitudes, Knowledge, and Behaviors of Physical Therapists Portuguese version questionnaire, consisted of 55 close-ended questions. It was analyzed response frequencies and associations between variables with logistic regression analyses. For the qualitative data, it was choose to perform semi-structured interviews in purposefully selected physical therapists to include different sociodemographic factors (especially those found to be statistically significant in the logistic regression) and survey responses regarding the physical therapists’ beliefs, attitudes, knowledge, and behaviors. The interviews were performed in an online software, where only audio contact was performed. The audios were anonymized and *verbatim* transcribed, and the texts explored by the thematic approach.

**Results:**

From the 277 physical therapists that shown interest in participating in the study, 193 fully completed the questionnaire and, from those, 10 participated in the interviews. The Portuguese physical therapists reported positive beliefs, attitudes, knowledge, and behaviors regarding EBP. Among the physical therapists characteristics it seems that age (younger therapists), education (participating in continuing education courses; belonging to practice-orientated organizations; having a doctorate degree; pursuing a higher academic degree; and being a clinical instructor), and workplace (working for someone else account; and academic sector) are the main factors in the Portuguese EBP implementation. The Portuguese physical therapists, beyond the physical therapists individual characteristics and workplace, also stated that evidence, patients, clinical experience, schools, country and physical therapy characteristics, may behave as facilitators or barriers when performing an EBP.

## Introduction

Evidence-Based Practice (EBP) is nowadays a widely used term by health-care practitioners. Originally developed at the McMaster Medical School in Canada in the 1980s, EBP can be defined as the conscientious, explicit and judicious use of current relevant available evidence combined with the health-care providers’ clinical expertise and the patients’ preferences, guiding clinical decisions about patients’ care (Hammell, 2001; Rosenberg & Donald, 1995; Sackett et al., 1996; Guyatt et al., 1992). Despite its importance (*e.g.*, improving the quality of healthcare), professional organizations identified it as a priority, and influential researchers and clinicians argue that health-care practitioners (such as Physical Therapists (PTs)) have an ethical obligation to base their practice on research findings, many still do not perform an EBP (Dannapfel, Peolsson & Nilsen, 2013). Personal, organizational, cultural, social, environmental, historical, educational, political, financial and demographic factors have being suggested as the main causers of this issue (Schreiber & Stern, 2005; Turner, 2001). As the profession is relatively new in Portugal (the first PT Portuguese school is from 1966–Escola Superior de Saúde de Alcoitão; Fonseca, 2012), currently little is known about the implementation of EBP in Portuguese PTs and the existing research is scarce to fully understand how the enumerated factors behave as a barrier and how to facilitate them (Schreiber & Stern, 2005; Zidarov, Thomas & Poissant, 2013). Therefore, if it could be understood the relationship between the different EBP “actors”, what are the barriers and how to avoid them, we could come closer to accomplishing one important goal (APTA, 2018): by 2020 PTs would be autonomous practitioners (*i.e.*, independent, self-determined, professional judgment and action) which, among other things, use an EBP.

So, the aim of this study is to know if the Portuguese PTs use an EBP, and collect and deeper understand the factors, barriers and facilitators associated with EBP.

## Materials & Methods

This study followed the Ethical Principles of the Helsinki Declaration (WMA, 2013) and was approved by the Porto University Faculty of Sport ethics committee (CEFADE24-2019).

This study incorporated a concurrent mixed-methods design (McPherson & Kayes, 2012; Creswell et al., 2011; Dixon-Woods et al., 2005; Driscoll et al., 2007; Sandelowski, Voils & Barroso, 2006; Schifferdecker & Reed, 2009; Jones et al., 2006), collecting quantitative (e-survey) and qualitative (semi-structured interviews) data to answer the research question. The study design flowchart is depicted in Fig. S1.

### Sample gathering

In an attempt to ensure the correct population sample, a national PT professional association–Associação Portuguesa de Fisioterapeutas (APFISIO)–e-mail database was requested for the Portugal PTs working class recruitment. Also, in an attempt to increase the number of enrolled participants, the e-mails of past students from 19 PT national schools were requested. According to the APFISIO, the number of participants would be around 12,000 PTs (APFISIO, 2016).

### Quantitative design

For the quantitative data, it was chosen to apply a self-administered e-survey. The e-survey was evaluated, designed, administered, conducted and collected according to established guidelines (Burns et al., 2008; Passmore et al., 2002; Sierles, 2003; Eysenbach, 2004).

The e-survey was e-mailed to all PTs in the APFISIO database in the regular online newsletter and to past PT students as a formal e-mail with a cover letter containing a small study’s information (background, justification and aims). Additionally, after reading the study’s information, the participants were invited to click in the e-survey link.

Before initiating the e-survey (in the front page), the purpose and context of the study, the data protection rights and how the results will be used (analyzed anonymously and confidentially, where the data gathered will only be used for statistical information in an academic environment), the criteria for selecting the participants and the reasons for non-participation, the possibility to terminate the e-survey at any time, instructions about how to fill and complete the e-survey, and the e-mail address for possible clarifications were explicitly stated. The consent for participation in the study was obtained through a informed consent statement. The e-survey was an adaptation of the Evidence-Based Practice: Beliefs, Attitudes, Knowledge, and Behaviors of Physical Therapists Portuguese version questionnaire (Ferreira et al., 2019), developed originally by Jette et al. (2003). The e-survey included 55 close-ended questions, 23 sociodemographic-related items and 32 EBP-related items. Sociodemographic items include a mix of personal, professional and academic data, and from the sociodemographic information, the PTs could not proceed to the next e-survey stage if they (Table 1):

**Table 1:** Inclusion and exclusion criteria.

Inclusion	Exclusion
have an active PT license;	do not have an active PT license or have another profession than PT;
obtained at least the PT bachelor degree;	obtained the PT bachelor degree in a foreign country;
work or have worked as a PT in the past 6 months in Portugal;	do not work in Portugal;
be able to read, write and speak Portuguese;	do not be able to read, write or speak Portuguese;
	be a PT bachelor student

Previously to sending the e-survey by e-mail, the e-survey was pre-tested by the authors and evaluated in its completion time, design, questions order, attractiveness, syntax, clarity, logic, correct question type and response format. Also, it was permitted to the respondents to review and change their answers. Using the Raosoft Sample Size Calculator (http://www.raosoft.com/samplesize.html), the sample size goal for this study was 373 responses, based in a 95% confidence level, a margin of error of 5% and a 50% response distribution (Singh & Masuku, 2014). To ensure that the sample size goal was achieved, after 2, 4 and 6 weeks respectively, a thank you note and a reminder containing the e-survey link was e-mailed.

### Qualitative design

Following the completion of the e-surveys, an individual qualitative strategy was performed. For the qualitative data collecting, it was chosen to apply semi-structured interviews with open-ended questions on the volunteer PTs. The interviews were conducted by one author, blinded to PTs characteristics and prior questionnaire answers, using Skype software (Microsoft Corporation, Rives de Clausen, Luxemburg), where only audio-recorded was performed–excluding any face-to-face or written contact. Furthermore, there was no relationship between the interviewers and the PTs prior to the study, and the interviewees were recruited by completing the study previous stages where, at the end of the e-survey, the respondents were invited to fill a space where a personal e-mail could be inserted. With this filling, they consent to be later contacted, if desired to continue to be studied. Following a review of questionnaire responses, the sample was purposefully selected to include different sociodemographic factors (especially those found to be statistically significant in the logistic regression) and survey responses regarding the PTs’ beliefs, attitudes, knowledge, and behaviors. To ensure a high participation rate, after 1, 2 and 4 weeks respectively, a thank you note, a reminder containing the interview objectives and a request to provide their most convenient dates for the interview, were e-mailed. The interview script had 12 core questions (Table S1), constructed according to Qu & Dumay (2011), and properly validated by an expert panel, before the interviews. The semi-structured interviews were performed according to Leech (2002) guidelines. However, because of the interviewees’ responses to specific questions, the number and order of questions was sometimes altered to a more in-deep investigation, to conserve the flow of the interview, and to maintain a positive relationship with the interviewee. Before initiating the core questions, an introductory section with the purpose of the study, the protection rights, how the data will be used, and some “warm-up” questions were included in order to build empathy and comfort. The “yes” or “no” answers were avoided. At the end of the core questions, the opportunity to the interviewees to add additional information and opinions that they found to be relevant to the thematic addressed was given. The interview script was tested on the first participant who, after the interview, was asked for feedback on the interview conduction, structure, design and phrasing of questions.

### Data analysis–quantitative

Response frequencies for the survey questions were determined and displayed in tabular and graphic formats, using the Microsoft Excel (Microsoft Corp, Redmond, Washington, DC, USA) and the IBM SPSS 26.0 (International Business Machines Corporation, Statistical Package for the Social Sciences, Armonk, NY, USA) software.

Before examining the associations between variables, some categories were collapsed in order to use them as dependent measures in logistic regression analyses. Using a similar strategy as Jette et al. (2003), for items with a four-point Likert scale the “Strongly Agree” and “Agree” categories were combined, as well as the “Strongly Disagree” and “Disagree” categories, so that responses fell into one of two categories: “Agree” or “Disagree”. For the items with a “Yes/No/Do Not Know” choice set, the “Do Not Know” category was combined with the “No” category, based on the belief that the lack of knowledge regarding EBP topics is, partially, similar as not performing it. For items categorized by the number of times, the lowest categories (≤1 and 2–5) were distinguished from the higher categories (6–10, 11–15 and ≥16), and combined in “Poor” for the lower values and “Good” for high values. For items that were designed to examine the degree of understanding of research terms, the “Understand Completely” and “Understand Somewhat” categories were combined so that a 2-category response was obtained: “Understand at Least Somewhat” or “Do Not Understand”. Lastly, in the barriers item, the PTs’ choices were collapsed into “Present” (if the PT chooses 1st, 2nd or 3rd) or “Absent” (no barrier choice).

For some of the sociodemographic data, where subsamples were smaller, we collapsed categories in an effort to derive stable models. For example, our sample included only 1 PT who indicated a professional Post-Doctorate degree, so we included him/her with others Philosophy Doctors (PhD) degrees. Furthermore, we combined the Certificate and Baccalaureate degrees into the same category (Baccalaureate), as in Portugal they are the minimum required professional entry-level.

After item categories were collapsed, logistic regression analyses were conducted to examine the associations with the PTs’ characteristics. It was used an α of 0.05 to define whether a model needed to be reported. Odds ratios (ORs) and their 95% CIs were determined for each level of the independent variables. CIs including 1.0 were considered as not statistically significant (McCormack, Vandermeer & Allan, 2013).

### Data analysis–qualitative

The data was analyzed with a Computer Assisted Qualitative Data Analysis Software, namely the NVivo v12 (QRS International, Doncaster, Victoria, Australia) (Welsh, 2002). The audios collected in the interviews were anonymized and *verbatim* transcribed. Then, the texts were explored by the six phases of thematic approach (Braun & Clarke, 2006). Data collection and analysis were continuously alternated in an iterative manner, in which three authors continuously reflected on, compared, discussed, and adjusted the codes and themes. The authors independently read the transcripts to obtain knowledge of the information gathered. Then, the authors reviewed the transcripts and identified the coding units in the transcripts. The researchers scrutinized the coding units and reviewed the text several times in order to add new codes to the previous ones. After, the coding units were combined into context units. The context units included several coding units and contained more than one quotation. The context units were merged into categories based in its similarity. The same process was performed in order to transform the categories in to system levels. Finally, all authors discussed the content of the categories using triangulating analysis with the FreeMind software (SourceForge Software). The discussions continued until no inconsistencies existed and a shared understanding was reached to prevent researchers’ bias and strengthen the internal validity. Then, the original classification tree was analyzed and further discussed with the expert panel, where some categories were collapsed, eliminated or renamed. Quotations were identified to report the findings and illustrate the content, and were translated from Portuguese to English. To ensure complete and transparent data reporting, the methodology was conducted according to established guidelines (Goodell, Stage & Cooke, 2016; DiCicco-Bloom & Crabtree, 2006; Wu, Wyant & Fraser, 2016; CASP, 2018; O’Brien et al., 2014; De Casterle et al., 2012; Tong, Sainsbury & Craig, 2007).

## Results

### Quantitative

**Figure 1:**
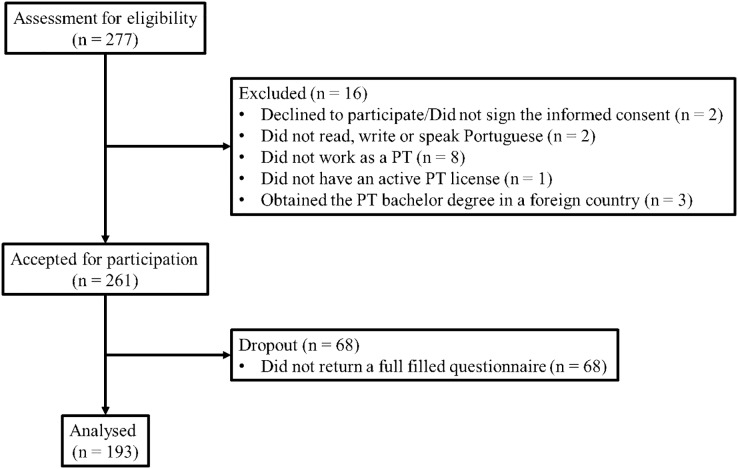
Questionnaire views, participation and completion.

From the 277 PTs that shown interest in participating in the study, only 193 (69.7%) fully completed the questionnaire (Fig. 1).

The average time to complete the e-survey was 12 min. The majority of the PTs that completed the questionnaire were females (73.1%), within the 30–39 years age group (44.6%), with a valid PT working license obtained between 5 to 10 years ago (32.1%). Furthermore, despite the majority only had the baccalaureate degree (61.1%), they shown interest in pursuing a higher academic degree in the future (66.3%). Most of them obtained the Certificate/Baccalaureate in the Escola Superior de Saúde de Alcoitão (20.7%) and were clinical instructors for PT students (51.3%). Additionally, they reported belonging to a practice-oriented organization (78.8%) and participate, at least once per year, in continuing education courses (89.1%). Regarding their practice, most of the PTs worked more than 40 h per week (44.6%), focusing their time taking care of patients (57.9%), leaving research and teaching as a low priority (87.6% and 82.4% within the 0–25% range, respectively). Typically, the PTs treated more than 15 adult patients per day, with orthopedic conditions (58%, 63.9% and 39.9%, respectively). The majority worked in Lisbon (35.2%), in an urban environment (82.9%), for someone else’s account, with 5 to 10 PTs, in the private sector (76.7%, 43.5% and 63.2%, respectively)–private clinics (21.2%). For a more detailed data analysis, the descriptive statistics of the PTs personal and practice characteristics are presented in Table 2.

**Table 2:** PTs personal and practice characteristics.

Characteristic	Frequency (%)	Characteristic	Frequency (%)
*Sex*		*Working Hours per Week*	
Male	52 (26.9%)	<20	5 (2.6%)
Female	141 (73.1%)	20–30	26 (13.5%)
*Age Groups*		31–40	76 (39.4%)
20–29 years	48 (24.9%)	>40	86 (44.6%)
30–39 years	86 (44.6%)	*Patients per Day*	
40–49 years	28 (14.5%)	1–5	29 (8.3%)
≥50 years	31 (16.1%)	6–10	71 (18.1%)
*Valid License*		11–15	49 (9.7%)
<5 years	27 (14%)	>15	44 (63.9%)
5–10 years	62 (32.1%)	*Number of PTs in the Facility*	
11–15 years	46 (23.8%)	0	46 (23.8%)
>15 years	58 (30.1%)	1–5	84 (43.5%)
*Degree*		6–10	27 (14%)
Certificate	4 (2.1%)	11–15	15 (7.8%)
Baccalaureate	118 (61.1%)	>15	21 (10.9%)
Master	58 (30.1%)	*Percentage of Total Work Time in:*	
Doctorate	12 (6.2%)	Patient Care	
Post-doctorate	1 (0.5%)	0%	2 (1%)
*Pursue a Higher Academic Degree*		5–25%	11 (5.7%)
Yes	128 (66.3%)	30–50%	17 (8.8%)
No	24 (12.4%)	55–75 %	52 (26.9%)
Do Not Know	41 (21.2%)	80–100 %	111 (57.9%)
*Participate in Continuing Education*		Researcher	
Yes	172 (89.1%)	0%	83 (43%)
No	21 (10.9%)	5–25%	86 (44.6%)
*Belong to a Practice-oriented Organization*		30–50%	19 (9.8%)
Yes	152 (78.8%)	55–75%	5 (2.6%)
No	41 (21.2%)	Teacher	
*Instructor*		0%	117 (60.6%)
Yes	99 (51.3%)	5–25%	42 (21.8%)
No	94 (48.7%)	30–50%	19 (9.8%)
*Certificate/Baccalaureate School*		55–75 %	10 (5.2%)
ESSATLA	12 (6.2%)	80–100 %	5 (2.6%)
ESSCVP	5 (2.6%)	*Location of the Facility*	
ESSUA	3 (1.6%)	Rural	11 (5.7%)
ESSL	5 (2.6%)	Suburban	21 (10.9%)
ESSP	18 (9.3%)	Urban	160 (82.9%)
ESSS	16 (8.3%)	Do not Treat Patients	1 (0.5%)
ESSA	40 (20.7%)	*Facility District*	
ESSVA	5 (2.6%)	Açores	5 (2.6%)
ESSVS	10 (5.2%)	Aveiro	14 (7.3%)
ESSLD	11 (5.7%)	Braga	8 (4.1%)
ESSEM	7 (3.6%)	Bragança	2 (1%)
ESSJP – Vila Nova de Gaia	4 (2.1%)	Castelo Branco	2 (1%)
ESSJP – Viseu	1 (0.5%)	Coimbra	14 (7.3%)
ESTeSC	28 (14.5%)	Évora	2 (1%)
ESTeSL	18 (9.3%)	Faro	4 (2.1%)
ISSAA	3 (1.6%)	Guarda	4 (2.1%)
UFP	7 (3.6%)	Leiria	11 (5.7%)
		Lisboa	68 (35.2%)
		Madeira	3 (1.6%)
		Portalegre	1 (0.5%)
		Porto	27 (14%)
		Santarém	5 (2.6%)
		Setúbal	13 (6.7%)
		Viana do Castelo	3 (1.6%)
		Vila Real	2 (1%)
		Viseu	4 (2.1%)
		Do Not Treat Patients	1 (0.5%)
		*Type of Facility*	
		Town Hall	1 (0.5%)
		Physical Medicine and Rehabilitation Center	28 (14.5%)
		Health Center	9 (4.7%)
		Geriatric Center/Resting Home	20 (10.4%)
		Private Clinic	41 (21.2%)
		Sports Club	3 (1.6%)
		Home Care	8 (4.1%)
		Commercial or Industrial Company	2 (1%)
		Healthcare Provider Company	2 (1%)
		Physical Therapy Office	18 (9.3%)
		Private Hospital	8 (4.1%)
		Public or Public-Private Partnership Hospital	31 (16.1%)
		Elementary or Secondary School	1 (0.6%)
		Higher Education Institute or Research Center	4 (2.1%)
		Continuing Care Unit	14 (7.3%)
		Other	2 (1%)
		Do Not Treat Patients	1 (0.5%)
		*Majority of Patients Condition*	
		Cardiovascular/pulmonary	16 (8.3%)
		Palliative Care	12 (6.2%)
		Hospital Health Care	6 (3.1%)
		Primary Health Care	2 (1%)
		Dermatological	1 (0.5%)
		Sport	5 (2.6%)
		Aging	28 (14.5%)
		Aquatic Physical Therapy	2 (1%)
		Orthopedic	77 (39.9%)
		Neurological	23 (11.9%)
		Pediatric	10 (5.2%)
		Women’s Health	8 (4.1%)
		Mental Health	1 (0.5%)
		Other	1 (0.5%)
		Do Not Treat Patients	1 (0.5%)
		*Majority of Patients Age Group*	
		Pediatric (≤18 years)	13 (6.7%)
		Adult (19–64 years)	112 (58%)
		Geriatric (≥65 years)	67 (34.7%)
		Do Not Treat Patients	1 (0.5%)
		*Work Sector*	
		Public	57 (29.5%)
		Private	122 (63.2%)
		Academic	14 (7.3%)
		*Work Modality*	
		Own Account	45 (23.3%)
		Someone Else’s Account	148 (76.7%)

**Note:**

Abbreviations: ESSATLA, Escola Superior de Saúde Atlântica; ESSCVP, Escola Superior de Saúde da Cruz Vermelha Portuguesa; ESSUA, Escola Superior de Saúde da Universidade de Aveiro; ESSL, Escola Superior de Saúde de Leiria; ESSP, Escola Superior de Saúde do Porto; ESSS, Escola Superior de Saúde de Setúbal; ESSA, Escola Superior de Saúde de Alcoitão; ESSVA, Escola Superior de Saúde do Vale do Ave; ESSVS, Escola Superior de Saúde do Vale do Sousa; ESSLD, Escola Superior de Saúde Dr. Lopes Dias; ESSEM, Escola Superior de Saúde Egas Moniz; ESSJP, Escola Superior de Saúde Jean Piaget; ESTeSC, Escola Superior de Tecnologia e da Saúde de Coimbra; ESTeSL, Escola Superior de Tecnologia e da Saúde de Lisboa; ISSAA, Instituto Superior da Saúde do Alto Ave; UFP, Universidade Fernando Pessoa.

**Figure 2:**
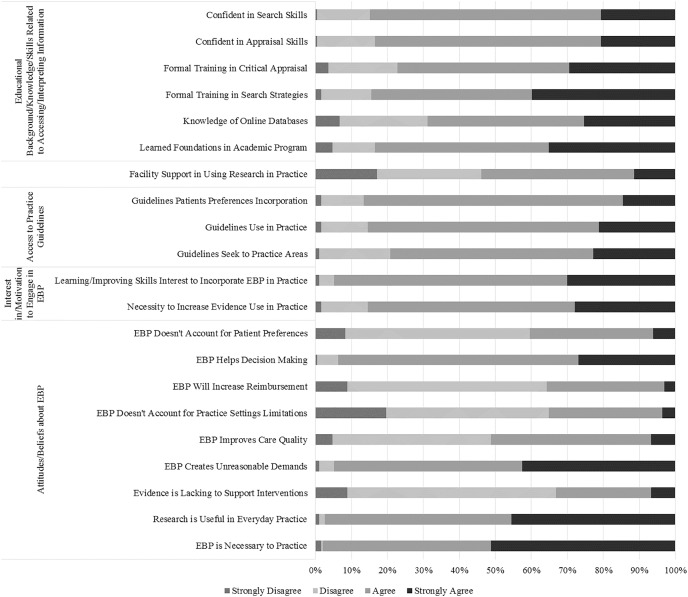
PTs’ choices in the attitudes and beliefs about EBP, interest in and motivation to engage in EBP, access to practical guidelines, access to and availability of information to promote EBP, and educational background, knowledge and skills related to accessing and interpreting information sub-groups.

Concerning the items sub-group Attitudes and Beliefs About EBP, respondents stated they held generally positive attitudes and beliefs regarding EBP. The only exception was in the item 32, where they Disagreed (45.1%) that EBP may increase their reimbursement. Moreover, there is the necessity to highlight the item 31 (“EBP does not take into account the limitations of my clinical practice setting”), where the PTs’ answers were very balanced between Agree (44.6%) and Disagree (44.0%). The respondents’ answers are presented in Fig. 2.

Regarding the statistically significant associations between PTs’ characteristics and their perceived Attitudes and Beliefs About EBP, therapists that participate at least once per year in continuing education courses were 2.9 and 3.7 more likely to agree that “EBP Doesn’t Account for Practice Settings Limitations” and that “Evidence is Lacking to Support Interventions” in comparison to those that do not participate in continuing education courses (*p* = 0.033 and *p* = 0.040, respectively). Also, PTs that treat 11 to 15 patients per day were 2.5 times more likely to agree that “EBP Doesn’t Account for Practice Settings Limitations”, in comparison with those who treated more than 15 patients per day (*p* = 0.036). Furthermore, PTs that belong to a practice-orientated organization were 4.1 and 4.2 more likely to agree that “EBP Improves Care Quality” and that “EBP Helps Decision Making” in comparison to those that do not belong to a practice-orientated organization (*p* = 0.033 and *p* = 0.019, respectively). Moreover, male PTs were 2.6 times more likely to agree that “EBP Will Increase Reimbursement” and 55% less likely to agree that “EBP Creates Unreasonable Demands” in comparison to their female peers (*p* = 0.004 and *p* = 0.034, respectively). Likewise, PTs that work by their own account were 91% less likely to agree that “EBP is Necessary to Practice” (*p* = 0.044). Table S2 summarizes the sub-group logistic regression information.

In the items sub-group Interest in and Motivation to Engage in EBP, they Agreed that they needed to increase the use of evidence in the daily practice (57.5%). The PTs’ characteristics that were more likely to agree were female therapists that worked in the public sector, for someone else account (OR 0.427 (95% CI [0.187–0.977]) for “Male” – *p* = 0.044; OR 3.012 (95% CI [0.909–9.977]) for “Public” – *p* = 0.009; and OR 0.401 (95% CI [0.172–0.936]) for “Own Account” – *p* = 0.035). Overall, the responders shown interest in learning or improving the skills necessary to incorporate EBP into practice (64.8%). The PTs’ characteristics that were more likely to agree with the overall findings were therapists that have a Certificate/Baccalaureate degree (OR 8.850 (95% CI [1.734–45.178]) – *p* = 0.009), that devote most of their time treating several patients (OR 0.089 (95% CI [0.010–0.786]) for “1–5 Patients Day” – *p* = 0.029; and OR 0.009 (95% CI [0.000–0.272]) for “0% Patient Care” – *p* = 0.007; OR 0.016 (95% CI [0.002–0.162]) for“5–25% Patient Care” – *p* = 0.000; OR 38.333 (95% CI [3.958–371.244]) for “0% Teacher” – *p* = 0.002; and OR 27.333 (95% CI [1.890–395.245]) for “5–25% Teacher” – *p* = 0.015) and do not work in the academic sector (OR 31.111 (95% CI [3.248–297.981]) for “Public” – *p* = 0.003; and OR 16.389 (95% CI [3.734–71.938]) for “Private” – *p* = 0.000) (Fig. 2 and Table S3).

**Figure 3:**
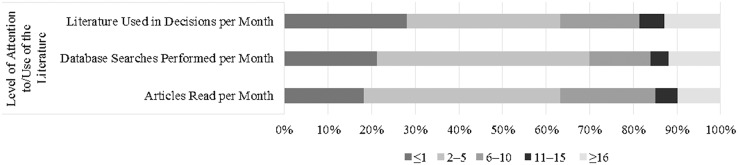
PTs’ choices in the level of attention to and use of the literature sub-group.

About the items sub-group Level of Attention to and Use of the Literature, PTs read, use and search a small number of professional literature (2–5 – 45.1%, 35.2% and 48.7%, respectively) (Fig. 3). Regarding the statistically significant associations between PTs’ characteristics and their Level of Attention to and Use of the Literature, male therapists were 2.1 to 2.4 times more likely to read, use and search professional literature in comparison to their female peers (*p* = 0.022, *p* = 0.010 and *p* = 0.022, respectively). Similarly, younger PTs were more likely to read and search professional literature in comparison to more experienced therapists (OR 4.105 (95% CI [1.556–10.830]) – *p* = 0.004; and OR 3.778 (95% CI [1.448–9.856]) – *p* = 0.007, respectively). In contrast, it was found that PTs with lower level academic degrees read and search less literature in comparison with Doctorate level peers (79% – *p* = 0.013 and 81% – *p* = 0.008, respectively). Furthermore, PTs that spend moderate time in patient care were more likely to read and search literature in comparison with PTs who spend 80–100% of their time treating patients (30–50% – OR 3.181 (95% CI [1.122–9.020]) (*p* = 0.030) and 55–75% – OR 3.054 (95% CI [1.533–6.085]) (*p* = 0.002) in “Articles Read per Month”; and 5–25% – OR 7.540 (95% CI [1.873–30.359]) (*p* = 0.004) and 30–50% – OR 6.786 (95% CI [2.201–20.922]) (*p* = 0.001) in “Databases Searches Performed per Month”). Similarly, PTs that spend moderate time as researchers were more likely to search literature in comparison with PTs who spend no time in that activity (OR 2.379 (95% CI [1.230–4.601]) – *p* = 0.010 for “5–25%”; and OR 6.825 (95% CI [2.294–20.306]) – *p* = 0.001 for “30–50%”). Still, PTs that belong to a practice-orientated organization were 2.5 times more likely to read more articles in comparison to those that do not belong to a practice-orientated organization (*p* = 0.030). Moreover, it was found that younger PTs were more likely to have good levels in professional literature searched in comparison to their older peers (OR 4.529 (95% CI [1.676–13.017]) – *p* = 0.005). Also, PTs that pursue a higher academic degree were more likely to search professional literature (OR 0.328 (95% CI [0.115–0.932]) – *p* = 0.036 for “No”; and OR 0.350 (95% CI [0.155–0.793]) – 0.012 for “Do Not Know”). Furthermore, those who work in the public sector were 80% less likely to search it in comparison to PTs who work primarily in the academic context (*p* = 0.011). Table S4 summarizes the sub-group logistic regression information.

**Figure 4:**
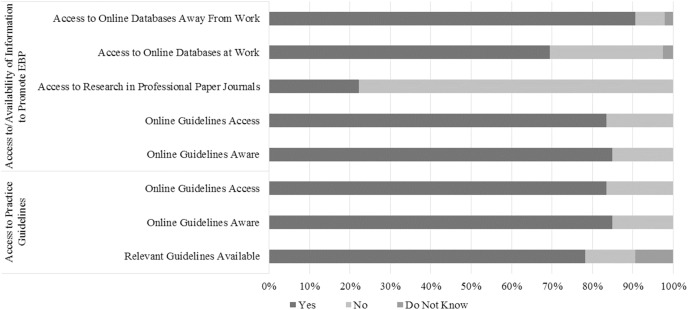
PTs’ choices in the access to practical guidelines, and access to and availability of information to promote EBP sub-groups.

Within the items sub-group Access to Practical Guidelines, most of the PTs know, seek, use, and access practice guidelines, and incorporate them with patient preferences (78.2%, 56.5%, 64.2%, 85%, 83.4% and 72%, respectively) (Figs. 2 and 4). Concerning the associations between PTs’ characteristics and the referred sub-group, clinical instructors were 66% less likely to think that guidelines are not available for topics related to their practice (*p* = 0.004) and were 3.1 time more likely to use them in their practice (*p* = 0.012). Additionally, PTs that pursue a higher academic degree and participate at least once a year in continuing education courses were more likely to use practice guidelines (OR 0.158 (95% CI [0.059–0.427]) – *p* = 0.000 for “No”; and OR 6.039 (95% CI [2.251–16.206]) – *p* = 0.000 for “Yes”, respectively). Furthermore, it was found that PTs that spend no time as teachers were 9.1 times more likely to be in agreement that practice guidelines incorporate patients’ preferences in comparison with those who spend 80–100% (*p* = 0.025). Table S5 summarizes the sub-group logistic regression information.

Regarding the items sub-group Access to and Availability of Information to Promote EBP, the majority of the respondents reported that, generally, they have access to EBP at work or at home. However, this access is mainly by the use of electronic databases (69.4% at work and 90.7% at home), since the majority assumed that they do not have access to current research through professional journals in their paper form (77.7%) (Fig. 4). From the different PTs’ characteristics, the ones that were more likely to assume that they do not have access to current research through professional journals in their paper form, include non-clinical instructors and therapists that spend less time in teaching (OR 0.369 (95% CI [0.179–0.763]) – *p* = 0.007 for “Yes”; and OR 25.250 (95% CI [2.651–240.495]) – *p* = 0.005 for “0%”, respectively). Additionally, male therapists were the ones who have an easier access to online databases at work (*p* = 0.018). Nevertheless, in general, PTs Agreed that the facility supports the use of research in practice (42.5%) (Fig. 2). From the different PTs’ characteristics, the ones who were more likely to agree include therapist that work 20–30 h per week (OR 5.250 (95% CI [1.669–16.517]) – *p* = 0.005), treating 6–10 patients per day (OR 2.350 (95% CI [1.087–5.079]) – *p* = 0.030), 30–50% as researcher (OR 6.963 (95% CI [1.884–25.736]) – *p* = 0.04), working by their own account (OR 3.444 (95% CI [1.623–7.310]) – *p* = 0.001), in either the academic or private work sector. (Table S6).

Concerning the items sub-group Educational Background, Knowledge and Skills Related to Accessing and Interpreting Information, most Agreed that they learned the foundations for EBP (48.2%), and receive training for finding and critically appraise research literature (43.5% and 47.7%, respectively), as part of their academic preparation. Furthermore, they were self-confident in their skills to critical appraisal and find research (62.7% and 64.2%, respectively). They were also familiar with medical search engines (44.6%). Figure 2 shows the distribution of the sub-group responses. Regarding the statistically significant associations between PTs’ characteristics and their Educational Background, Knowledge and Skills Related to Accessing and Interpreting Information, the oldest therapists were less likely to agree that they learned their foundations for EBP and had formal training in search strategies, in comparison to younger therapists (20–29 – OR 9.474 (95% CI [2.397–37.436]) (*p* = 0.001) and 30–39 – OR 3.547 (95% CI [1.395–9.015]) (*p* = 0.008) in “Learned Foundations”; and 20-29 – OR 5.262 (95% CI [1.941–14.260]) (*p* = 0.001) and 30–39 – OR 3.577 (1.521–8.410) (*p* = 0.003) in “Search Strategies”). Similar comparison was found for formal training in critical appraisal, where PTs in the 20–29 and in the 30–39 age group were 4.2 and 2.6 times more likely to agree in contrast with the older therapists (*p* = 0.008 and *p* = 0.037, respectively). As a complementation, PTs that had less years of license report a similar pattern as younger therapists in the “Formal Training in Search Strategies” (OR 3.832 (95% CI [1.276–11.508]) – *p* = 0.017 for “<5”; and OR 3.283 (95% CI [1.475–7.307]) – *p* = 0.04 for “5–10”). In contrast, more experienced therapists were more likely to agree that they were confident in search skills (OR 0.254 (95% CI [0.078–0.824]) – *p* = 0.023 for “5–10”; and OR 0.267 (95% CI [0.078–0.916]) – *p* = 0.036 for “11–15”). Still, clinical instructors were 3.3 and 2.3 times more confident in searching and critically reviewing professional literature, in comparison to non-clinical instructors (*p* = 0.008 and *p* = 0.039, respectively). Additionally, not knowing if the PT wants to pursue a higher academic degree had a negative impact in learning his foundations for EBP as part of the academic preparation, received formal training in search strategies, and help them to be more familiar with medical search engines, since therapists who pursue it are more likely to agree (OR 0.286 (95% CI [0.122–0.699]) – *p* = 0.004; OR 0.370 (95% CI [0.177–0.772]) – *p* = 0.008; and OR 0.233 (95% CI [0.092–0.541]) – *p* = 0.001, respectively). Additionally, PTs that work in the Public and Private sectors were 4.7 and 7.1 times more likely to agree that “I learned the foundations for EBP as part of my academic preparation”, in comparison with the academic sector peers (*p* = 0.015 and *p* = 0.001, respectively). In the same item, therapists that are not teachers were 13.1 times more likely to agree, in contrast with PTs that are teachers (*p* = 0.007). Table S7 summarizes the sub-group logistic regression information.

**Figure 5:**
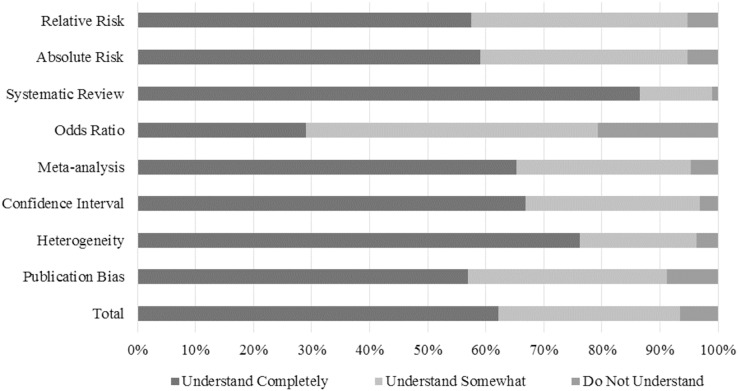
PTs’ scientific terms understanding.

The positive findings in the Educational Background, Knowledge and Skills Related to Accessing and Interpreting Information sub-group was further confirmed, as most the PTs Understand Completely (62.2%) the presented scientific terms. Nevertheless, the most understood scientific term was “Systematic Review” (17.4%) and the least was “Odds Ratio” (9.7% in Understand Somewhat and 39.6% in Do Not Understand). The only term that had statistically significant associations was “Publication Bias”. The PTs who do not understand the term were non-clinical instructors working by their own account (OR 0.262 (95% CI [0.082–0.836]) – *p* = 0.024 for “Clinical Instructor”; and OR 3.339 (95% CI [1.205–9.252]) – *p* = 0.020 for “Own Account”, respectively). The understanding levels of the terms associated with EBP are described in Fig. 5 and the sub-group logistic regression information is summarized in Table S8.

**Figure 6:**
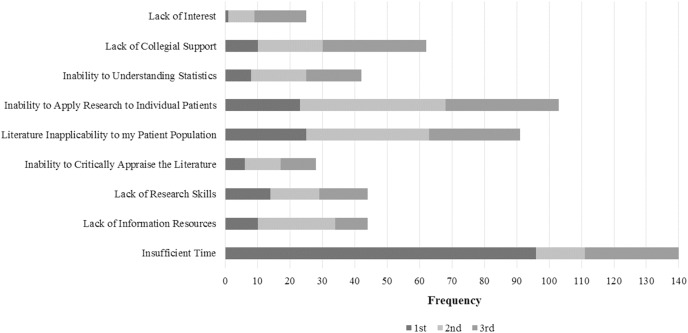
PTs’ choices in perceived barriers to EBP use.

Within the items sub-group Perceived Barriers for Evidence Using in Practice, the PTs reported “Insufficient time” as the most important barrier (24.2%), followed by “Inability to apply research findings to individual patients with unique characteristics” (17.8%), and finally “Lack of generalizability of the literature findings to my patient population” (15.7%). In the ranking, “Insufficient time” was the most chosen for 1st (49.7%), and “Inability to apply research findings to individual patients with unique characteristics” for 2nd and 3rd (23.3% and 18.1%, respectively). The barriers distribution is exhibited in Fig. 6. Regarding the statistically significant associations between PTs’ characteristics and their perceived barriers, PTs that work by their own account were 2.4 more likely to choose “Lack of information resources” and “Lack of research skills” as barriers, in comparison with other therapists that work for someone else account (*p* = 0.022). At the same time, they were 62% less likely to choose “Lack of collective support among my colleagues in my facility” as a barrier, in comparison with their peers (*p* = 0.022). Less experienced PTs were more likely to choose “Insufficient Time” as a barrier in comparison with more experienced therapists (OR 3.106 (95% CI [1.031–9.356]) – *p* = 0.044 for “<5”; and OR 2.941 (95% CI [1.297–6.668]) – *p* = 0.010 for “5-10”). Furthermore, non-clinical female therapists’ instructors were more likely to choose “Insufficient Time” in comparison with their male clinical instructors peers (*p* = 0.029 and *p* = 0.016, respectively). It was also found that the number of PTs in the facility might influence the “Inability of Apply Research to Individual Patients” barrier, as in facilities with more than 15 PTs, this barrier was less chosen (OR 3.491 (95% CI [1.096–11.124]) – *p* = 0.0034 for “0”; OR 4.945 (95% CI [1.654–14.790]) – *p* = 0.004 for “1–5”; and OR 12.800 (95% CI [2.545–64.372]) – *p* = 0.002 for “11–15”). Additionally, PTs that treat 1–5 patients per day were 72% less likely to choose the “Lack of Collegial Support” in comparison with the ones that treat more than 15 patients per day (*p* = 0.041). Finally, differences were found between public and academic work sector, as academic therapists were more likely to have restrictions to apply research to individual patients (OR 0.271 (95% CI [0.076–0.986]) – *p* = 0.044 for “Public”) (Table S9).

For a more detailed data analysis, the descriptive statistics of the PTs EBP responses are presented in Table S10.

### Qualitative

From the 193 PTs that completed the e-survey, only 67 (34.7%) volunteered for the interviews. From those, 23 PTs were selected, but only 10 responded to the emails. The participants’ characteristics in the qualitative study are outlined in Table 3.

**Table 3:** PTs’ characteristics in the qualitative study.

Characteristics	Physical therapists									
	PT 1	PT 2	PT 3	PT 4	PT 5	PT 6	PT 7	PT 8	PT 9	PT 10
Sex	Male	Female	Female	Male	Male	Female	Male	Female	Female	Female
Age	20–29	20–29	20–29	40–49	<50	30–39	30–39	30–39	20–29	30–39
Years of License	>5	>5	>5	<15	<15	11–15	5–10	5–10	5–10	11–15
Academic Degree	Bac.	Bac.	Bac.	PhD	Mas.	Bac.	Mas.	Mas.	Bac.	Bac.
Clinical Instructor	No	No	No	No	No	No	Yes	Yes	No	No
Working Hours	31–40	20–30	20–30	<40	<40	31–40	<40	31–40	<40	31–40
Patients Day	6–10	6–10	11–15	6–10	1–5	11–15	6–10	11–15	11–15	6–10
PTs in the Facility	1–5	1–5	1–5	<15	0	1–5	0	6–10	6–10	0
% Time in:										
*Patient Care*	80–100%	55–75%	55–75%	30–50%	5–25%	80–100%	55–75%	30–50%	80–100%	80–100%
*Researcher*	5–25%	5–25%	30–50%	5–25%	5–25%	0%	5–25%	5–25%	0%	5–25%
*Teacher*	0%	0%	0%	55–75%	80–100%	0%	30–50%	5–25%	0%	0%
Work Sector	Private	Private	Public	Academic	Academic	Private	Private	Public	Private	Private
Work Mode	Others Account	Own Account	Own Account	Others Account	Others Account	Others Account	Own Account	Others Account	Others Account	Own Account

**Notes:**

All include PTs Pursue a Higher Academic Degree, Participate in Continuing Education Courses and Belong to a Practice-Orientated Organization.

Abbreviations: Bac., Baccalaureate; Mas., Master; PhD, Philosophy Doctor.

The interviews were made from January to April 2020. At the end, 313 minutes of recordings were obtained (31 minutes in average – 21 minimum [FT 3]; 72 maximum [FT 5]), which generated 71 transcript pages (7 average – 5 minimum; 13 maximum). The interviews offered compelling fragments of PT’s experiences, opinions and beliefs about evidence-based practice. In most cases, the qualitative data underpins the survey findings. The most often spoken word by PTs was “persons”, followed by “evidence” and “practice” (191 times, 179 times and 128 times, respectively). The Fig. S2 shows the word cloud frequency.

With the interviews, six main themes were identified: EBP definition; EBP concept origin; Main actors and their individual importance; Relations between the main actors; EBP in the workplace; EBP national wide. The classification tree is illustrated in Fig. S3.

**Figure 7:**
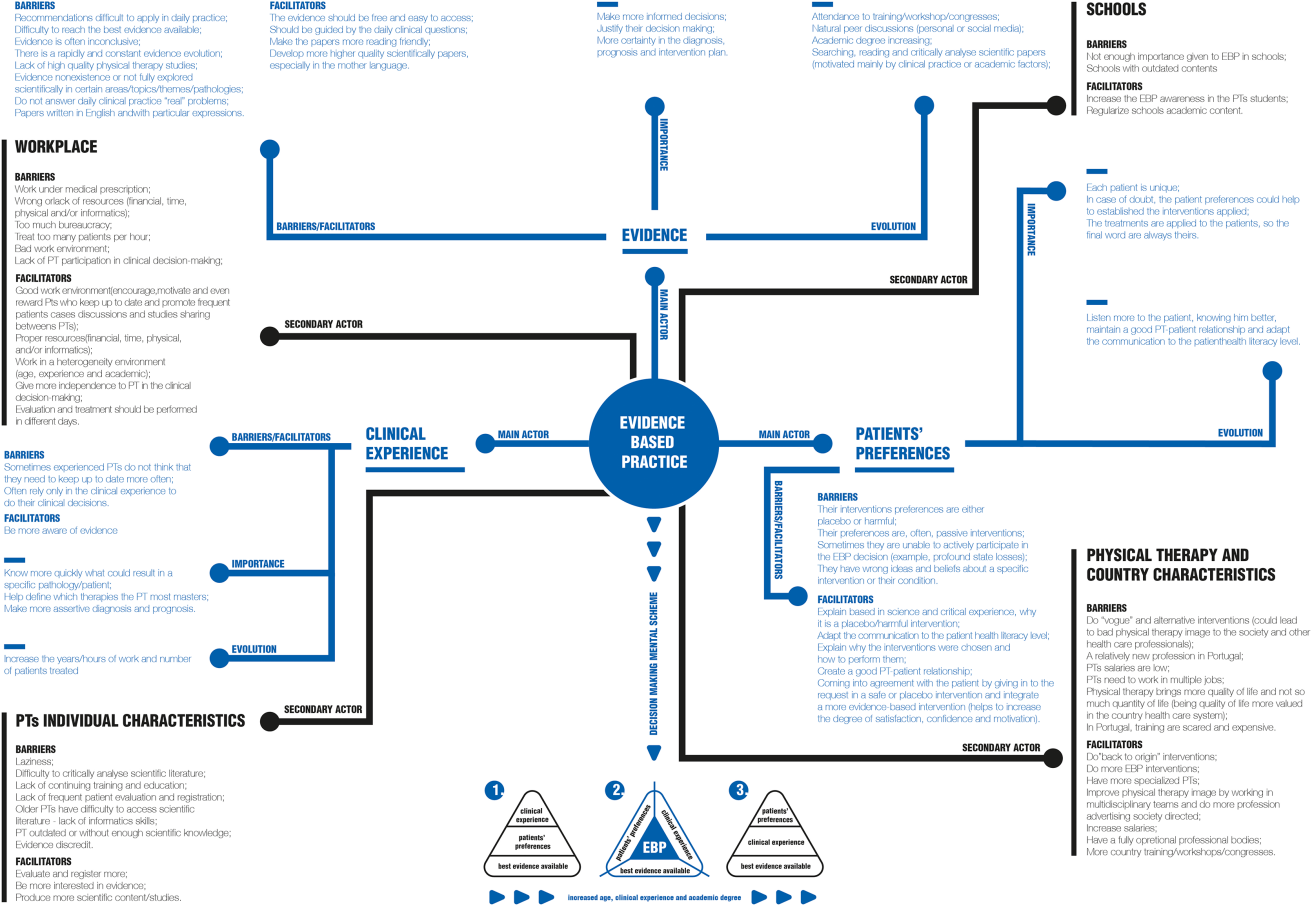
Qualitative conceptual map.

The overall findings and their conceptual map are present in the Fig. 7. Also, the interviews qualitative analysis and the respective quotations are present in the Supplemental Material.

## Discussion

The aim of this study was to know if the Portuguese PTs use an EBP, to collect and deeper understand the factors, barriers and facilitators associated to EBP. The overall results shown that the Portuguese PTs have positives beliefs, attitudes, knowledge and behaviors regarding EBP. Most of the PTs answer Agree or Yes to the questionnaire questions, except in the item “Application of EBP is necessary in the practice of physical therapy”, where the majority of the PTs answered Strongly Agree. There were some negative items such as “I have access to current research through professional journals in their paper form”, “EBP does not take into account patient preferences”, “My reimbursement rate will increase if I incorporate EBP into my practice”, “The adoption of EBP places an unreasonable demand on PTs”, and “Strong evidence is lacking to support most of the interventions I use with my patients”, where the answers were Disagree or No. Still, there was the item “EBP does not take into account the limitations of my clinical practice setting” where the answers balanced between Agree (44.6%) and Disagree (44.0%). Despite the answers to these items were negative, they shown progressive attitudes regarding EBP, as disagreeing to the majority of these statements is considered as positive (Bernhardsson et al., 2014). The only item that may be evaluated as truly negative is not having access to professional journals in their paper form. This was also confirmed in the qualitative data, where almost none of the interviewed PTs had access to information this way. Quantitative data shown that, nowadays, PTs access evidence through professional online databases, as presented in the sub-group Access to and Availability of Information to Promote EBP responses. Other forms to keep up to date stated included: natural peer discussions; increase academic degree; and the attendance to trainings/workshops/congresses. So, it seems that, for the Portuguese PTs, EBP is easily spread by either personal contact or electronic information, swapping from the traditional paper form. This change was also found in other countries, especially after the introduction and access to computers and the Internet (Haines & Silagy, 2001). However, this change could be problematic for older PTs as they could not have enough informatics skills to do an EBP. In fact, Bridges, Bierema & Valentine (2007) found that age and years licensed as a PT were negatively correlated with the propensity to adopt EBP, as older PTs may not know how to access the scientific literature. For the Portuguese PTs, and as expected (Jones et al., 2006), EBP should include clinical experience, patients’ preferences and scientific evidence as main actors. Although some PTs balanced the main actors and others rank their importance, for all of them evidence was a key element in EBP, being mainly referred as the “best evidence available at the moment”. This definition is similar to the one used by Sackett et al. (1996). Despite its importance to make more informed decisions, to justify the intervention plan, and to make more assertive diagnostics and prognostics, one barrier is to gather the best recent evidence, as evidence is rapidly and constantly evolving. For example, in August 2019, PEDro indexed 44,309 articles (34,619 trials, 9,004 reviews, and 686 guidelines), and the number is predicted to double by 2025 (Moseley et al., 2020). Nevertheless, nowadays the task of searching for relevant information is greatly aided by the Internet and the existence of very sophisticated search engines and databases (Haines & Silagy, 2001). It seems that the majority of the Portuguese PTs are confident in their search skills and aware of online databases, as they Agree in both items, being guidelines, systematic reviews, meta-analyses, RCTs and expert opinion papers the most common studies searched. From those, the PTs preferred reading from what is in higher level in the evidence hierarchy, such as guidelines, meta-analysis, systematic reviews or reviews. These choices are supported by evidence and associated with positive attitudes regarding EBP (Jones et al., 2006; Haines & Silagy, 2001; Harris et al., 2014; Dijkers, Murphy & Krellman, 2012). Moreover, it was found that PTs preferred online access to research summaries or systematic reviews to save time, to filter and critique research articles (Salbach et al., 2011). However, as older PTs are less informatics educated and in their school time online databases did not exist, they may not have the necessary abilities to search in electronic databases and reach the most recent evidence. Jette et al. (2003) found similar associations, where younger PTs have reported more confidence in skills than the elders, due to the fact that they are part of a generation that grew up with computers and Internet at school and at home. As suggested by the interviewed PTs, this issue could be overcame with a work environment with age heterogeneity, where older PTs could give information about their clinical experiences and, in return, younger PTs could show how to search in online databases. Knowledge exchange between peers (informal–such as, everyday conversations about a specific intervention; and formal–such as, meetings in order to reflect on research), has been demonstrated as an important factor in modeling EBP, as PTs reported that their peers are the first they turn to when they need more information, a second opinion about a intervention, or to obtain support for testing a new method (Dannapfel, Peolsson & Nilsen, 2013; Kloda & Bartlett, 2009). For example, in the Iles & Davidson (2006) study, 42% of the responders confirmed that they formally shared and discussed evidence with others in their department or practice. Additionally, in the Nilsagård & Lohse (2010) study, 38% of the PTs mentioned colleagues as sources of information related with EBP. Individuals tend to be linked to others who are close to them in physical distance and who are relatively homophilous in social characteristics (Hoffmann, Probst & Christinck, 2007). So, this strategy, could be a good facilitator for work and evidence associated barriers. Another related factor is being a clinical instructor. In our qualitative data whenever there was a statistically significant association, those who are clinical instructors shown more positive beliefs, attitudes, knowledge and behaviors regarding EBP. So, as clinical instructors are usually older PTs (20–29 years 31%, 30–39 years 54%, 40–49 years 64% and ≥50 years 67%), the clinical instructor position may “force” PTs to be more aware of EBP. Salbach et al. (2010) also found that clinical instructors PTs were more likely to use research in practice, compared with non-clinical instructors.

Additionally, increasing the academic degree may have a positive influence in EBP. The advantages pointed out by the PTs were better search and critical analyze literature skills, as for the lectures and dissertation it is needed to constantly base their options on the most recent and higher quality evidence and to perform studies. In our quantitative data, Baccalaureate degree PTs were 9 times more likely to Agree that “I am interested in learning or improving the skills necessary to incorporate EBP into my practice” and were 80% less likely to search and read studies per month, in comparison to PhD PTs. Moreover, for the oldest PT interviewed, increasing the academic degree was very important to do an EBP. This is evidenced in the Nilsagård & Lohse (2010), Grimmer-Somers et al. (2007), Alshehri et al. (2017), and Bridges, Bierema & Valentine (2007) studies where higher-level degrees PTs (Master’s 2 years–PhD) had greater overall values in knowledge, behaviors, attitudes, adoption, awareness and prerequisites, compared with their lower-level degree peers.

Another suggested facilitator was having frequent trainings/courses/workshops provided by professional bodies. It could not only be a good way to receive summarized evidence and have the possibility to be in contact with new treatments, but it could also exist more specialized workshops or lectures to give older PTs the necessary skills to search in online databases. Evidence has been showing that directed educational meetings have a positive impact in the EBP (Dannapfel, Peolsson & Nilsen, 2013; Schreiber & Stern, 2005; Bridges, Bierema & Valentine, 2007; Van der Wees et al., 2008; Menon et al., 2009; Dizon, Grimmer-Somers & Kumar, 2012; Prior, Guerin & Grimmer-Somers, 2008). For example, courses and in-service training were reported to be the two most important methods of keeping up to date for UK PTs (Stevenson, Lewis & Hay, 2004). Moreover, in a study with American PTs (Fruth et al., 2010), after EBP presentations given in the workplace, most of them reported gaining new information and integration of the material, increased the beliefs, attitudes, knowledge and behaviors regarding EBP, and even welcome additional presentations. Furthermore, in our study, PTs that belong to a professional practice-oriented organization use and have more positive attitudes and beliefs regarding EBP, comparing to their peers that do not belong to it. However, not only in the PTs opinion Portuguese trainings/workshops/congresses are scarce and expensive for the national panorama, but also, PTs also think that older PTs do not have enough positive attitudes and believes regarding EBP (as they are lazy, do not give enough weigh on evidence in their daily clinical practice, and do not do continuous training). This last statement was more referred by younger PTs as, in their opinion, older PTs plan their sessions based only in clinical experience. Despite clinical experience may help PTs to know quicker what could result in a specific condition, to help define which interventions they master most, and to make more assertive diagnostics and prognostics, in the PTs point of view, as in evidence more information could be gathered about a topic comparing with a lifetime work, practice should be mainly evidence guided. This practice change may be one of the most important factors related with PTs EBP (Schreiber & Stern, 2005). Moreover, such as any knowledge, clinical experience can be biased and poorly used (Jones et al., 2006). It is common to find that people have a misinterpretation of their ability, skills, performance and/or knowledge (Kruger & Dunning, 1999). What separates the experienced practitioners from the experts is the wise application of their clinical experience (Schreiber & Stern, 2005). Also, these professionals have a better ability to “sense” when something is wrong, known as intuition (Schreiber & Stern, 2005; Welsh & Lyons, 2001). So, although having frequent trainings/workshops/congresses could help older PTs (for example, PTs that regularly participate in continuing education courses were 6 times more likely to use guidelines in practice), currently this may not be the most correct facilitator for this population, as referred by one of the oldest interviewed PT: “I can tell you that I have colleagues who graduated like me in 1987 and I never saw them in a congress”. Nevertheless, as suggested by the PTs, if the national professional bodies demand that for keeping the physical therapy license, PTs have to show/prove that they keep updating (such as, attending to congresses), this may be a good incentive to solve this lack of interest. In American PTs (Landers et al., 2005), it was found that in states with mandatory continuing education, PTs have a clinical practice beneficial effect, compared with their peers in non-mandatory continuing education states. Other stated incentive may be from the health care units where the PTs work, as the attendance to trainings/workshops/congresses or increasing academic degree should be encouraged, motivated and even rewarded. However, the PTs are pessimistic on being rewarded (at least financially), as in the quantitative data the majority do not think that their reimbursement rate will increase if they incorporate EBP into their practice.

Other factors that may help to decrease the evidence interest for older PTs include: evidence is often inconclusive or in certain areas non-existent; lack of high quality physical therapy studies; recommendations are difficult to apply in practice and do not respond to the clinical practice “real” problems; most of the papers are written in English and difficult to read/understand. Similar evidence-related barriers were reported in other studies (Schreiber & Stern, 2005; Dijkers, Murphy & Krellman, 2012; Salbach et al., 2007; Hannes et al., 2009; Maher et al., 2004; Francke et al., 2008; Herbert et al., 2001; Metcalfe et al., 2001; Morris, 2003; Ramírez-Vélez et al., 2015; Silva, Costa & Costa, 2015; Dao et al., 2018; Vongsirinavarat et al., 2020; Funabashi, Warren & Kawchuk, 2012). However, some of these barriers could be hard to overcome. For instance, as papers in the native language are published in national journals (and those may have a small impact factor), scientists do not tend to publish in these journals because it gives them fewer academic credits, pursuing international English-written journals with superiors impact factors (Mathew effect–“the rich get richer”) (Hannes et al., 2009; Villanueva et al., 2020). Furthermore, to have the “best evidence available at the moment”, besides gathering evidence, it is needed to critically analyze it (Rosenberg & Donald, 1995). For the majority of the Portuguese PTs population this may not be an issue as most Agree that “I am confident in my ability to critically review professional literature”, the majority of the scientific terms were “Understand Completely” and the barrier “Poor ability to critically appraise the literature” was only chosen 4.8%. However, in addition to the already exposed barriers, older PTs may not have the necessary skills to search, critically analyze and be aware of EBP in school. Therefore, this may explain why differences were found between ages in “I learned the foundations for EBP as part of my academic preparation”, “I have received formal training in search strategies for finding research relevant to my practice”, and “I received formal training in critical appraisal of research literature as part of my academic preparation” items and other beliefs, attitudes, knowledge and behaviors regarding EBP explored in the qualitative data. Likewise, in the Salbach et al. (2007) study respondents with less than 5 years of experience were 31.2 times more likely to have learned the foundations of EBP in their academic preparation, 9.3 times more likely to report having received formal training with search strategies and 99.8 times more likely to report having received formal training in critical appraisal skills, compared with respondents who had more than 15 years of practice experience. Additionally, Australians PTs (Iles & Davidson, 2006) showed similar behaviors, as the mean score of recent graduates was 4.95 points higher than experienced, regarding overall EBP skills, and Brazilians PTs (Silva, Costa & Costa, 2015) who had graduated within 9 years had more knowledge and skills compared with those who had graduated more than 9 years ago in: EBP experience in graduate or postgraduate degree (*p* = 0.001); sufficient EBP knowledge in the graduate or postgraduate degree (*p* = 0.004); EBP core elements understanding (p = 0.004); ability to critically analyze a scientific paper (*p* = 0.005); and access online databases frequency (*p* = 0.009). Jette et al. (2003) pointed out that the more likelihood of finding positive beliefs among younger and more recently licensed respondents (compared with their older peers) suggests a more recent focus on the EBP topic within physical therapy education programs.

Although it seems that Portuguese physical therapy schools are getting better in preparing their students for EBP, it was also recommended to regularize and update their academic contents, and continuing to insist in EBP contents until doing EBP becomes almost a habit for the PTs. This facilitator was identified as early as 2005 (Schreiber & Stern, 2005) and reinforced by the qualitative data gathered by Rotor et al. (2020) where, in the PT students views, their EBP education had a positive impact on EBP knowledge and attitudes.

Other researchers found, as we did, that the primary barrier to implement EBP is lack of time (Schreiber & Stern, 2005; Jette et al., 2003; Bernhardsson et al., 2014; Bridges, Bierema & Valentine, 2007; Iles & Davidson, 2006; Nilsagård & Lohse, 2010; Grimmer-Somers et al., 2007; Fruth et al., 2010; Salbach et al., 2007; Francke et al., 2008; Metcalfe et al., 2001; Vongsirinavarat et al., 2020; Cranney et al., 2001; Barnard & Wiles, 2001; Ballin et al., 1980; Gorgon et al., 2013; Da Silva et al., 2015; Scurlock-Evans, Upton & Upton, 2014; Yahui & Swaminathan, 2017; Akinbo et al., 2008; Queiroz & dos Santos, 2017; Alrowayeh et al., 2019; Cobo-Sevilla et al., 2018) and, although not directly evidence-related, it was found that “Insufficient time” might also influence the PTs updating ability, as they could not have sufficient time to do, search and read scientific literature. In fact, such as in Ballin et al. (1980) and in Jette et al. (2003) studies, besides “Insufficient time”, the most pointed EBP barriers in the questionnaire were patient-evidence-related: “Lack of generalizability of the literature findings to my patient population” and “Inability to apply research findings to individual patients with unique characteristics” (16% and 18%, respectively). “Insufficient time” barrier could be lessened if: evidence is present in their facilities in a form of a lecture (Fruth et al., 2010); research evidence be more easy to access, time efficient, and relevant to practice (Bridges, Bierema & Valentine, 2007); more national guidelines are accessible (Woolf et al., 1999; Gagliardi, 2012). Moreover, this could be more problematic for younger and less experienced PTs where statistical significant associations were found between years of license and the barrier. This was further pointed out in the qualitative data as the interviewed PTs stated that, since salaries are low in Portugal, PTs (especially in a younger age) need to have multiple jobs and work for several hours to have a minimal decent standard living. So, as suggested, increasing the PTs salaries could have a positive influence in using EBP, since Portuguese PTs may began to choose working in one workplace, freeing time for other tasks, namely do, search and read scientific literature. For example, in a study with Americans PTs (Ballin et al., 1980), when asked to select the single most significant inducer to stimulate research, the most frequent choice was salary increase (31%).

Although no statistically significant association was found, “Insufficient time” was further related in the qualitative data with workplace, especially in Physical Therapy Medicine and Rehabilitation Centers. In those workplaces, the PTs pointed out that there are excessive patients “treated” per hour (it was referred 10 patients/hour), sometimes the work is very bureaucratic (time is more spent in filling paper forms and less in treating patients), and the PTs have to respect what is prescribed by the physiatrist (the PTs do not actively participate in the intervention plan and generally interventions are chosen by what is stated-founded). Belgians PTs reported similar barriers, but still added that some doctors are influenced by commercial firms that promote certain products, prejudicing an EBP (Hannes et al., 2009). So, as a basic approach to influence behavior is through work environmental change and with the provision of new structures or resources (Dannapfel, Peolsson & Nilsen, 2013; Schreiber & Stern, 2005; Francke et al., 2008; Ballin et al., 1980; Glanz, Rimer & Viswanath, 2008), the PTs suggested that: the number of patients treated per hour needs to be reduced (recommending one patient per hour); it is needed to give more autonomy to the PTs and more participation in the intervention plan decision-making; treatments and evaluations should be performed in different sessions; it is needed to move away for what is stated-founded and focusing in interventions that are evidence-based; and having more and correct resources. Additionally, despite not directly related with this workplace, statistically significant associations were found in work sector, work mode, and the total time spent in patient care, research and teaching. However, the best characteristic related to EBP in each group could not be reached.

An explanation for the barriers may relay on the image that other health professional and the Portuguese society have regarding physical therapy. The bad image could be from: some discredit and disrespect of the Portuguese PTs on using an EBP (some PTs still do not do a practice evidence-based or use “vogue” and alternative interventions); the profession focuses mainly on providing quality of life and not quantity of life (being quantity of life more valued in the current national health system); the number of PTs involved in top health policy makers is still scarce (there is not even a full functional physical therapy national professional bodies yet); the profession in Portugal is considered as new (54 years); and the fullness of its work areas is unknown (association still exists with manual therapy and masseuses). Related barriers were also reported by Belgians PTs (Hannes et al., 2009). There were suggested facilitators that may overcome these issues, such as: working in multidisciplinary teams; doing more evidence-based and physical therapy related interventions in daily practice; evaluate and register more; increasing the participation in scientific studies; having more specialized PTs or increasing the academic degrees; and doing more physical therapy advertising and actions society-centered.

As showed in the qualitative data, for the interviewed PTs, EBP is not a “fixed” concept. This was already shown in the literature, where other factors were added to the early EBP definition proposed by Sackett et al. (1996). As in our study, Lizarondo, Grimmer-Somers & Kumar (2011), and Dijkers, Murphy & Krellman (2012), added social, cultural, economic, political and work environment, as important influencing factors in EBP. Nevertheless, our study found a paradigm shift in the EBP concept. As discussed, it seems that age, clinical experience and academic degree may have an important role in clinical practice. So, it seems that the most important influence in EBP is personal-related factors. Our data shown that, as younger PTs are less experienced, they only have evidence to guide them in the clinical decision-making. In contrast, older and more experienced PTs may not have the skills to search in online databases and critically analyze literature, so they do their clinical decision-making based mainly in their clinical experience. Moderate old and experienced PTs may be the ones that have the necessary skills to do an EBP, balancing evidence with clinical experience. Furthermore, increasing academic degree may help PTs achieving better performances in search and critically appraise scientific literature, reaching the best evidence. Patients’ preferences are complex and the level of importance given to it may have other factors to influence it (Francke et al., 2008; Haynes, Devereaux & Guyatt, 2002; Cruz, Moore & Cross, 2012). In one hand, patients’ preferences could establish the interventions applied in case of doubt, in other hand, their preferences, habits, attitudes and beliefs could be a barrier to EBP (sometimes they have a wrong idea about their condition and their interventions preferences may be either placebo or harmful). To overcome this barrier and to have a successful intervention plan, a good relationship, trust and proper communication PT-patient is mandatory (Haynes, Devereaux & Guyatt, 2002). As older and more experienced PTs in their lifetime had to deal with more patients (with different personalities), they could increase their PT-patients interaction skills (Barnard & Wiles, 2001). Additionally, undergraduate students and novice therapists tend to focus on patients’ symptoms, impairments, and functional problems, instead of integrating patients’ problems with their needs, life styles and environment (Cruz, Moore & Cross, 2012). However, this could not be confirmed in our study, as in the qualitative data experienced and less experienced PTs shown similar PT-patients interaction skills. This may be explained by the PTs preparation in Portuguese schools for patient care (Caeiro, Cruz & Pereira, 2014). Nevertheless, since the PT is more experienced and aware of the best recent evidence, he/she has the responsibility to clarify the patient. Furthermore, as each patient is unique, the PT should adapt the communication to the patient’s health literacy level (Schipper et al., 2015; Opsommer & Schoeb, 2014). In fact, in a study with Kuwaiti PTs (Alrowayeh et al., 2019), 90% confirmed that the key factor, when selecting and applying the best therapy, is their capability to effectively communicate with the patient. Therefore, often a good, simple, correct, informed and assertive communication could be enough for information assimilation (Schipper et al., 2015; Opsommer & Schoeb, 2014). To ensure a better communication, both oral and written informations should be given to the patient (Hoffmann, Probst & Christinck, 2007; Glanz, Rimer & Viswanath, 2008; Schipper et al., 2015). Furthermore, patient education is usually in the guidelines as a core treatment (Zadro, O’Keeffe & Maher, 2019).

## Conclusions

In conclusion, the Portuguese PTs reported positive beliefs, attitudes, knowledge, and behaviors regarding EBP. Among the PTs characteristics it seems that age (younger therapists), education (participating in continuing education courses; belonging to practice-orientated organizations; having a doctorate degree; pursuing a higher academic degree; and being a clinical instructor), and workplace (working for someone else account; and academic sector) are the main factors in the Portuguese EBP implementation. The Portuguese PTs, beyond the PTs individual characteristics and workplace, also stated that evidence, patients, clinical experience, schools, country and physical therapy characteristics, may behave as facilitators or barriers when performing an EBP.

There were found some limitations in our study. One limitation was the number of valid questionnaires. From the potential 12,000 Portuguese physical therapists only 1.6% returned the completed questionnaire, the minimal 373 sample size goal was not reached and 26% of the questionnaires were incomplete. This is may be due to the questionnaire extension and complexity. As no reward was given to the responders PTs, they may felt tired after answering some of the questions. Additionally, although the questionnaire was anonymous, as many of the questions were clinical practice related, some of the PTs may not felt comfortable to answer them. Moreover, as the questionnaire has 32 questions EBP related, the sociodemographic questions should have appeared after the EBP related questions, since the PTs, even if tired, could be more enthusiastic in answering more individual questions, possibly increasing the number of complete questionnaires. Additionally, after combining quantitative and qualitative data, we also believe that the questionnaire may be revise, in an attempt to approximate to the Portuguese reality (*e.g.*, adding the language factor to the barriers). Another factor may be the PTs lack of interest in participating in national studies. Although the quantitative data shows that the Portuguese PTs have positive beliefs, attitudes, knowledge and behaviors regarding EBP, in our qualitative data all the PTs interviewed agreed that EBP was still not widely practiced in Portugal. So, it is possible that therapists with more positive attitudes, or those who are more confident in their evidence-based practice knowledge and practice, are more likely to return surveys and could correspond to the majority of the PTs sample (Iles & Davidson, 2006). For example, in our study, the PTs that pursued a higher academic degree, regularly participate in continuing education courses, and belong to a professional practice-oriented organization were in greater number compared with their peers (66%, 89%, and 79%, respectively). These characteristics contributed to better beliefs, attitudes, knowledge and behaviors regarding EBP. Therefore, the results could not truly represent the Portuguese PTs practice. Person-to-person questionnaire and direct clinical practice observations may solve some of these limitations. For example, differences were found in treatments use between surveys completed by PTs and audits of clinical notes, in musculoskeletal conditions (Zadro, O’Keeffe & Maher, 2019). In surveys, 54% of PTs chose recommended treatments, 43% chose not recommended treatments, and 81% chose treatments that have no recommendation. Based on audits of clinical notes, 63% of patients received recommended physical therapy-delivered treatments, 27% received not recommended treatments, and 45% received treatments that have no recommendation. The sample size limitation could also influence the logistic regression analyses. It is expected that if the sample size was enlarged, the number and strength of significant logistic regressions would also increase.

## Supplemental Information

10.7717/peerj.12666/supp-1 Supplemental Information 1 Instructions for Submissions Methodological flowchart.

10.7717/peerj.12666/supp-2 Supplemental Information 2 Instructions for Submissions Word cloud.

10.7717/peerj.12666/supp-3 Supplemental Information 3 Classification tree.

10.7717/peerj.12666/supp-4 Supplemental Information 4 Instructions for Submissions Semi-Structured Interviews Script.

10.7717/peerj.12666/supp-5 Supplemental Information 5 Logistic regression tables. Instructions for Submissions ^a^ Nagelkerke R^2^; ^b^ In logistic regression, one level of the independent variable serve as reference against which the odds of the other levels occurring are determined.

10.7717/peerj.12666/supp-6 Supplemental Information 6 Instructions for Submissions EBP Questionnaire responses. Instructions for Submissions a–Relative risk; b–Absolute risk; c–Systematic review; d–Odds ratio; e–Meta-analysis; f–Confidence interval; g–Heterogeneity; h–Publication bias; α–Insufficient time; β–Lack of information resources; γ–Lack of research skills; δ–Poor ability to critically appraise the literature; ε–Lack of generalizability of the literature findings to my patient population; θ–Inability to apply research findings to individual patients with unique characteristics; σ–Lack of understanding of statistical analysis; ψ–Lack of collective support among my colleagues in my facility; ω–Lack of interest; NA–Not Applicable.

10.7717/peerj.12666/supp-7 Supplemental Information 7 Quotations.

10.7717/peerj.12666/supp-8 Supplemental Information 8 Quantitative. Raw Data-SPSS

## Additional Information and Declarations

### Data Availability

The following information was supplied regarding data availability: The raw measurements are available in the Supplemental Files.
