# Supplementary figures and images for: Measuring evidence-based practice in physical therapy: a mix-methods study
Source: PeerJ. 2022 Jan 4;10:e12666. doi: 10.7717/peerj.12666 (PMC8740513; doi:10.7717/peerj.12666)

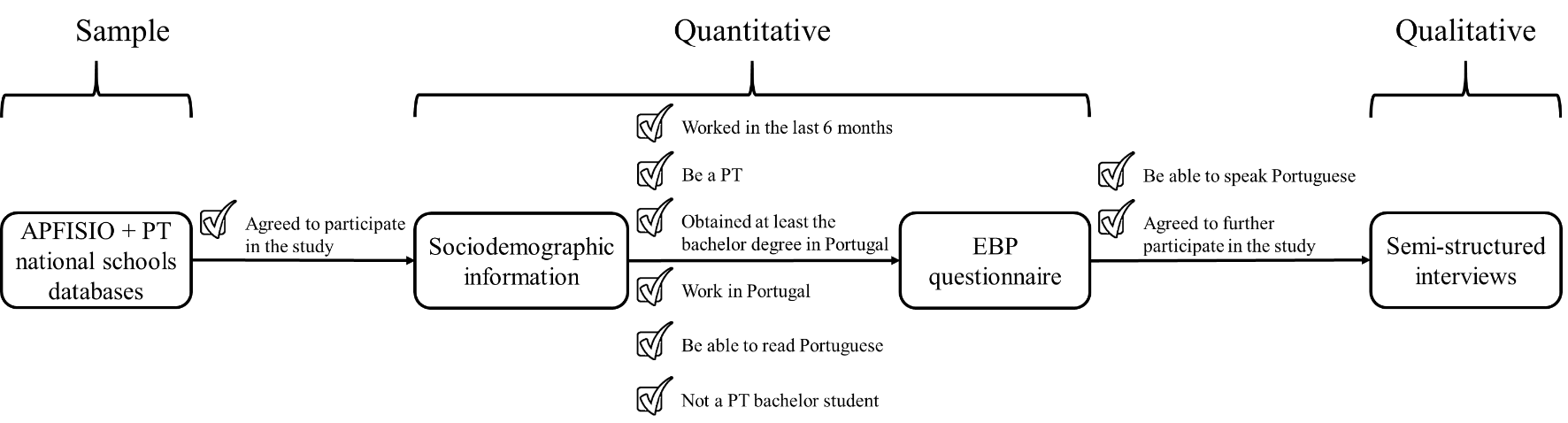

Supplement: Supplemental Information 1 [file peerj-10-12666-s001.png]

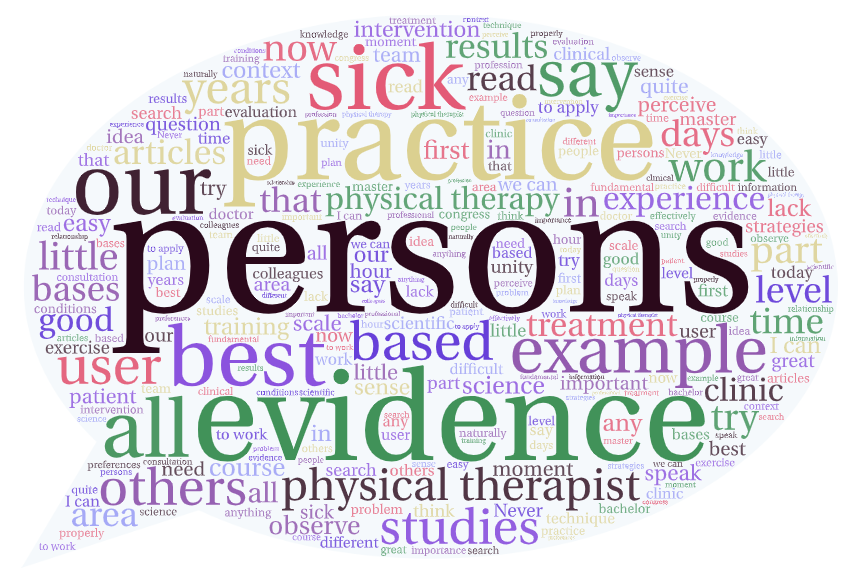

Supplement: Supplemental Information 2 [file peerj-10-12666-s002.png]

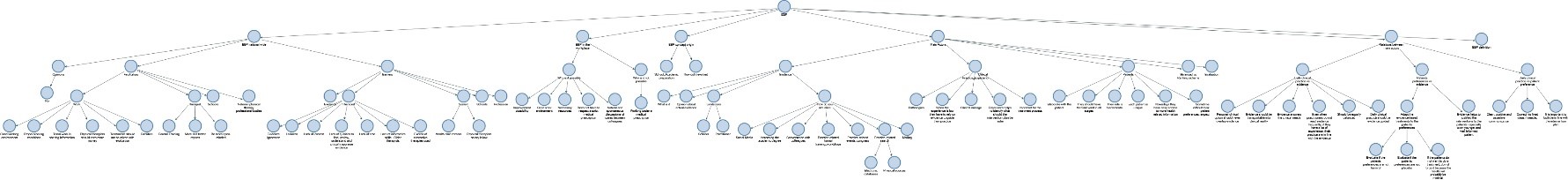

Supplement: Supplemental Information 3 [file peerj-10-12666-s003.jpg]
