# Supplementary material for: Measuring evidence-based practice in physical therapy: a mix-methods study
Source: PeerJ. 2022 Jan 4;10:e12666. doi: 10.7717/peerj.12666 (PMC8740513; doi:10.7717/peerj.12666)
Supplement: Supplemental Information 4 [file peerj-10-12666-s004.docx]

Table 1 – Semi-Structured Interviews Script

| 1. Why did you choose to be a physical therapist? 2. What is your opinion about the physical therapist profession’s valorization in Portugal?    1. Do you think we could improve the profession’s valorization?       1. If so, why/in which way (examples)?       2. If not, why? 3. Do you know the ***evidence-based practice*** concept?    1. Could you explain what evidence-based practice is for you?    2. Do you know its prepositions, ideas and main actors?       1. If so, from your point of view, how are they related? 4. Which was your first contact with the evidence-based practice concepts? 5. As a physical therapist, what is your opinion about evidence-based practice? 6. Do you feel motivated for an evidence-based practice? 7. Is evidence-based practice applicable in your daily clinical practice?    1. If so, why?    2. If not, why? 8. For you, what is the patient's role in an evidence-based practice? 9. What is your opinion about the current evidence regarding the most common pathologies that you treat (examples/specific conditions)?    1. And about interventions (examples/specific conditions)? 10. Do you consider yourself an updated person regarding the scientific evidence in your area?     1. If so, which is the reason/motivation/frequency?        1. How do you keep yourself up to date?           1. Do you usually use guidelines?              1. If so, which is the reason/motivation/frequency?              2. If not, why?        2. Do you think the evidence influences your daily clinical practice?           1. If so, in which way?           2. If not, why?     2. If not, why?        1. For you, what strategies would work to improve your update? 11. In the context of an evidence-based practice, how do you perceive scientific knowledge and the clinical experience/practice?     1. Do you think that these two concepts are related?        1. How do they relate?        2. What strategies would you use to improve this relationship? 12. Do you think that you have adequate resources at your disposal for an evidence-based practice?     1. If so, what is the reason?        1. Can you give some examples?     2. If not, what are the main barriers for a non-evidence-based practice?        1. According to the examples you mentioned, what facilitating strategies do you suggest? |
| --- |
