# Supplementary material for: Measuring evidence-based practice in physical therapy: a mix-methods study
Source: PeerJ. 2022 Jan 4;10:e12666. doi: 10.7717/peerj.12666 (PMC8740513; doi:10.7717/peerj.12666)
Supplement: Supplemental Information 5 — Instructions for Submissions a Nagelkerke R2; b In logistic regression, one level of the independent variable serve as reference against which the odds of the other levels occurring are determined. [file peerj-10-12666-s005.docx]

Table 2 – Association Between PTs’ Characteristics and Their Attitudes and Beliefs About EBP

| **Attitude or Belief**  **(Agree)** | **Factor - Level** | **Odds Ratio (95% CI)** | ***P*** | ***R*^2 a^** |
| --- | --- | --- | --- | --- |
| EBP is Necessary to Practice |  |  |  |  |
|  | *Work Mode* |  | *0.044* | *0.137* |
|  | Own Account | 0.095 [0.010; 0.940] |  |  |
|  | Someone Else Account | Reference^b^ |  |  |
| EBP Creates Unreasonable Demands |  |  |  |  |
|  | *Sex* |  | *0.034* | *0.035* |
|  | Male | 0.445 [0.211; 0.941] |  |  |
|  | Female | Reference^b^ |  |  |
| EBP Improves Care Quality |  |  |  |  |
|  | *Belong to a Professional Practice-orientated Organization* |  | *0.033* | *0.066* |
|  | Yes | 4.083 [1.122; 14.864] |  |  |
|  | No | Reference^b^ |  |  |
| EBP Doesn’t Account for Practice Settings Limitations |  |  |  |  |
|  | *Participated in Continuing Education Courses* |  | *0.033* | *0.034* |
|  | Yes | 2.943 [1.090; 7.945] |  |  |
|  | No | Reference^b^ |  |  |
|  | *Patients day* |  | *0.015* | *0.074* |
|  | 1-5 | 1.348 [0.526; 3.454] | 0.534 |  |
|  | 6-10 | 0.713 [0.334; 1.524] | 0.383 |  |
|  | 11-15 | 2.483 [1.063; 5.796] | 0.036 |  |
|  | >15 | Reference^b^ |  |  |
| EBP Will Increase Reimbursement |  |  |  |  |
|  | *Sex* |  | *0.004* | *0.059* |
|  | Male | 2.634 [1.369; 5.067] |  |  |
|  | Female | Reference^b^ |  |  |
| Evidence is Lacking to Support Interventions |  |  |  |  |
|  | *Participated in Continuing Education Courses* |  | *0.040* | *0.038* |
|  | Yes | 3.736 [1.059; 13.174] |  |  |
|  | No | Reference^b^ |  |  |
| EBP Helps Decision Making |  |  |  |  |
|  | *Belong to a Professional Practice-orientated Organization* |  | *0.019* | *0.072* |
|  | Yes | 4.171 [1.269; 13.714] |  |  |
|  | No | Reference^b^ |  |  |

^a^ Nagelkerke R^2^; ^b^ In logistic regression, one level of the independent variable serve as reference against which the odds of the other levels occurring are determined.

Table 3 – Association Between PTs’ Characteristics and Their Interest in and Motivation to Engage in EBP.

| **Interest or Motivation (Agree)** | **Factor - Level** | **Odds Ratio (95% CI)** | ***P*** | ***R*^2 a^** |
| --- | --- | --- | --- | --- |
| Necessity to Increase Evidence Use in Practice |  |  |  |  |
|  | *Sex* |  | *0.044* | *0.036* |
|  | Male | 0.427 [0.187; 0.977] |  |  |
|  | Female | Reference^b^ |  |  |
|  | *Work Sector* |  | *0.032* | *0.064* |
|  | Public | 7.361 [1.655; 32.748] | 0.009 |  |
|  | Private | 3.012 [0.909; 9.977] | 0.071 |  |
|  | Academic | Reference^b^ |  |  |
|  | *Work Mode* |  | *0.035* | *0.039* |
|  | Own Account | 0.401 [0.172; 0.936] |  |  |
|  | Someone Else Account | Reference^b^ |  |  |
| Learning/Improving Skills Interest to Incorporate EBP in Practice |  |  |  |  |
|  | *Academic Degree* |  | *0.029* | *0.089* |
|  | Baccalaureate | 8.850 [1.734; 45.178] | 0.009 |  |
|  | Master | 5.500 [0.969; 31.222] | 0.054 |  |
|  | Doctorate | Reference^b^ |  |  |
|  | *Patients Day* |  | *0.008* | *0.174* |
|  | 1-5 | 0.089 [0.010; 0.786] | 0.029 |  |
|  | 6-10 | 0.802 [0.071; 9.118] | 0.859 |  |
|  | 11-15 | 1.116 [0.068; 18.387] | 0.939 |  |
|  | >15 | Reference^b^ |  |  |
|  | *Patients Care* |  | *0.002* | *0.287* |
|  | 0% | 0.009 [0.000; 0.272] | 0.007 |  |
|  | 5-25% | 0.016 [0.002; 0.162] | 0.000 |  |
|  | 30-50% | 0.145 [0.009; 2.443] | 0.180 |  |
|  | 55-75% | 0.148 [0.015; 1.463] | 0.102 |  |
|  | 80-100% | Reference^b^ |  |  |
|  | *Teacher* |  | *0.006* | *0.233* |
|  | 0% | 38.333 [3.958; 371.244] | 0.002 |  |
|  | 5-25% | 27.333 [1.890; 395.245] | 0.015 |  |
|  | 30-50% | 3.556 [0.405; 31.233] | 0.253 |  |
|  | 55-75% | 2.667 [0.250; 28.438] | 0.417 |  |
|  | 80-100% | Reference^b^ |  |  |
|  | *Work Sector* |  | *0.000* | *0.225* |
|  | Public | 31.111 [3.248; 297.981] | 0.003 |  |
|  | Private | 16.389 [3.734; 71.938] | 0.000 |  |
|  | Academic | Reference^b^ |  |  |

^a^ Nagelkerke R^2^; ^b^ In logistic regression, one level of the independent variable serve as reference against which the odds of the other levels occurring are determined.

Table 4 – Association Between PTs’ Characteristics and Their Attention to and Use of the Literature.

| **Attention an Use of Literature (Good)** | **Factor - Level** | **Odds Ratio (95% CI)** | ***P*** | ***R*^2 a^** |
| --- | --- | --- | --- | --- |
| Articles Read per Month |  |  |  |  |
|  | *Sex* |  | *0.022* | *0.037* |
|  | Male | 2.133 [1.115; 4.081] |  |  |
|  | Female | Reference^b^ |  |  |
|  | *Years of License* |  | *0.011* | *0.083* |
|  | < 5 | 4.105 [1.556; 10.830] | 0.004 |  |
|  | 5-10 | 1.051 [0.492; 2.248] | 0.897 |  |
|  | 11-15 | 0.809 [0.348; 1.881] | 0.622 |  |
|  | > 15 | Reference^b^ |  |  |
|  | *Academic Degree* |  | *0.039* | *0.049* |
|  | Baccalaureate | 0.209 [0.061; 0.720] | 0.013 |  |
|  | Master | 0.292 [0.080; 1.061] | 0.061 |  |
|  | Doctorate | Reference^b^ |  |  |
|  | *Belong to a Professional Practice-orientated Organization* |  | *0.030* | *0.037* |
|  | Yes | 2.449 [1.093; 5.490] |  |  |
|  | No | Reference^b^ |  |  |
|  | *Patients Care* |  | *0.014* | *0.089* |
|  | 0% | 2.828 [0.171; 46.683] | 0.467 |  |
|  | 5-25% | 2.356 [0.668; 8.308] | 0.182 |  |
|  | 30-50% | 3.181 [1.122; 9.020] | 0.030 |  |
|  | 55-75% | 3.054 [1.533; 6.085] | 0.002 |  |
|  | 80-100% | Reference^b^ |  |  |
| Literature Used in Decisions per Month |  |  |  |  |
|  | *Sex* |  | *0.010* | *0.047* |
|  | Male | 2.402 [1.232; 4.682] |  |  |
|  | Female | Reference^b^ |  |  |
| Database Searches Performed per Month |  |  |  |  |
|  | *Sex* |  | *0.022* | *0.037* |
|  | Male | 2.133 [1.115; 4.081] |  |  |
|  | Female | Reference^b^ |  |  |
|  | *Age* |  | *0.023* | *0.070* |
|  | 20-29 | 4.529 [1.676; 13.017] | 0.005 |  |
|  | 30-39 | 2.011 [0.741; 5.461] | 0.170 |  |
|  | 40-49 | 3.125 [0.976; 10.005] | 0.055 |  |
|  | ≥ 50 | Reference^b^ |  |  |
|  | *Years of License* |  | *0.024* | *0.068* |
|  | < 5 | 3.778 [1.448; 9.856] | 0.007 |  |
|  | 5-10 | 1.311 [0.614; 2.797] | 0.485 |  |
|  | 11-15 | 0.875 [0.374; 2.047] | 0.759 |  |
|  | > 15 | Reference^b^ |  |  |
|  | *Academic Degree* |  | *0.009* | *0.069* |
|  | Baccalaureate | 0.186 [0.054; 0.643] | 0.008 |  |
|  | Master | 0.361 [0.100; 1.307] | 0.121 |  |
|  | Doctorate | Reference^b^ |  |  |
|  | *Pursue a Higher*  *Academic Degree* |  | *0.009* | *0.071* |
|  | No | 0.328 [0.115; 0.932] | 0.036 |  |
|  | Do Not Know | 0.350 [0.155; 0.793] | 0.012 |  |
|  | Yes | Reference^b^ |  |  |
|  | *Patients Care* |  | *0.001* | *0.134* |
|  | 0% | 2.828 [0.171; 46.683] | 0.467 |  |
|  | 5-25% | 7.540 [1.873; 30.359] | 0.004 |  |
|  | 30-50% | 6.786 [2.201; 20.922] | 0.001 |  |
|  | 55-75% | 1.915 [0.954; 3.847] | 0.068 |  |
|  | 80-100% | Reference^b^ |  |  |
|  | *Researcher* |  | *0.002* | *0.109* |
|  | 5-25% | 2.379 [1.230; 4.601] | 0.010 |  |
|  | 30-50% | 6.825 [2.294; 20.306] | 0.001 |  |
|  | 55-75% | 0.788 [0.083; 7.489] | 0.835 |  |
|  | 0% | Reference^b^ |  |  |
|  | *Work Sector* |  | *0.032* | *0.051* |
|  | Public | 0.198 [0.057; 0.687] | 0.011 |  |
|  | Private | 0.348 [0.110; 1.102] | 0.073 |  |
|  | Academic | Reference^b^ |  |  |

^a^ Nagelkerke R^2^; ^b^ In logistic regression, one level of the independent variable serve as reference against which the odds of the other levels occurring are determined.

Table 5 – Association Between PTs’ Characteristics and Their Access to Practical Guidelines.

| **Access to Practical Guidelines** | **Factor - Level** | **Odds Ratio (95% CI)** | ***P*** | ***R*^2 a^** |
| --- | --- | --- | --- | --- |
| Relevant Guidelines Available (No) |  |  |  |  |
|  | *Clinical Instructor* |  | *0.004* | *0.071* |
|  | Yes | 0.339 [0.163; 0.703] |  |  |
|  | No | Reference^b^ |  |  |
| Guidelines Use in Practice (Agree) |  |  |  |  |
|  | *Pursue a Higher*  *Academic Degree* |  | *0.001* | *0.113* |
|  | No | 0.158 [0.059; 0.427] | 0.000 |  |
|  | Do Not Know | 0.814 [0.272; 2.438] | 0.713 |  |
|  | Yes | Reference^b^ |  |  |
|  | *Participated in Continuing Education Courses* |  | *0.000* | *0.104* |
|  | Yes | 6.039 [2.251; 16.206] |  |  |
|  | No | Reference^b^ |  |  |
|  | *Clinical Instructor* |  | *0.012* | *0.063* |
|  | Yes | 3.074 [1.281; 7.378] |  |  |
|  | No | Reference^b^ |  |  |
| Guidelines Patients’ Preferences Incorporation (Agree) |  |  |  |  |
|  | *Teacher* |  | *0.011* | *0.120* |
|  | 0% | 9.083 [1.321; 62.446] | 0.025 |  |
|  | 5-25% | 2.833 [0.404; 19.873] | 0.295 |  |
|  | 30-50% | 2.500 [0.306; 20.453] | 0.393 |  |
|  | 55-75% | 1.000 [0.112; 8.947] | 1.000 |  |
|  | 80-100% | Reference^b^ |  |  |

^a^ Nagelkerke R^2^; ^b^ In logistic regression, one level of the independent variable serve as reference against which the odds of the other levels occurring are determined.

Table 6 – Association Between PTs’ Characteristics and Their Access to and Availability of Information to Promote EBP.

| **Access and Availably of Information** | **Factor - Level** | **Odds Ratio (95% CI)** | ***P*** | ***R*^2 a^** |
| --- | --- | --- | --- | --- |
| Access to Research in Professional Paper Journals (No) |  |  |  |  |
|  | *Clinical Instructor* |  | *0.007* | *0.060* |
|  | Yes | 0.369 [0.179; 0.763] |  |  |
|  | No | Reference^b^ |  |  |
|  | *Teacher* |  | *0.005* | *0.131* |
|  | 0% | 25.250 [2.651; 240.495] | 0.005 |  |
|  | 5-25% | 8.923 [0.906; 87.840] | 0.061 |  |
|  | 30-50% | 8.667 [0.790; 95.088] | 0.077 |  |
|  | 55-75% | 6.000 [0.478; 75.344] | 0.165 |  |
|  | 80-100% | Reference^b^ |  |  |
| Access to Online Databases at Work (No) |  |  |  |  |
|  | *Sex* |  | *0.018* | *0.046* |
|  | Male | 0.381 [0.172; 0.845] |  |  |
|  | Female | Reference^b^ |  |  |
| Facility Support in Using Research in Practice (Agree) |  |  |  |  |
|  | *Working Hours* |  | *0.008* | *0.102* |
|  | < 20 | 3.818 [0.410; 35.570] | 0.239 |  |
|  | 20-30 | 5.250 [1.669; 16.517] | 0.005 |  |
|  | 31-40 | 0.773 [0.416; 1.445] | 0.414 |  |
|  | > 40 | Reference^b^ |  |  |
|  | *Patients Day* |  | *0.006* | *0.088* |
|  | 1-5 | 2.280 [0.865; 6.007] | 0.095 |  |
|  | 6-10 | 2.350 [1.087; 5.079] | 0.030 |  |
|  | 11-15 | 0.697 [0.304; 1.598] | 0.394 |  |
|  | > 15 | Reference^b^ |  |  |
|  | *Researcher* |  | *0.013* | *0.088* |
|  | 5-25% | 1.649 [0.898; 3.029] | 0.107 |  |
|  | 30-50% | 6.963 [1.884; 25.736] | 0.004 |  |
|  | 55-75% | 5.222 [0.559; 48.775] | 0.147 |  |
|  | 0% | Reference^b^ |  |  |
|  | *Work Sector* |  | *0.000* | *0.147* |
|  | Public | 0.158 [0.043; 0.570] | 0.005 |  |
|  | Private | 0.709 [0.210; 2.395] | 0.580 |  |
|  | Academic | Reference^b^ |  |  |
|  | *Work Mode* |  | *0.001* | *0.078* |
|  | Own Account | 3.444 [1.623; 7.310] |  |  |
|  | Someone Else Account | Reference^b^ |  |  |

^a^ Nagelkerke R^2^; ^b^ In logistic regression, one level of the independent variable serve as reference against which the odds of the other levels occurring are determined.

Table 7 – Association Between PTs’ Characteristics and Their Educational Background, Knowledge and Skills Related to Accessing and Interpreting Information.

| **Education, Skill or Knowledge (Agree)** | **Factor - Level** | **Odds Ratio (95% CI)** | ***P*** | ***R*^2 a^** |
| --- | --- | --- | --- | --- |
| Learned Foundations in Academic Program |  |  |  |  |
|  | *Age* |  | *0.005* | *0.114* |
|  | 20-29 | 9.474 [2.397; 37.436] | 0.001 |  |
|  | 30-39 | 3.547 [1.395; 9.015] | 0.008 |  |
|  | 40-49 | 3.789 [1.052; 13.652] | 0.042 |  |
|  | ≥ 50 | Reference^b^ |  |  |
|  | *Pursue a Higher*  *Academic Degree* |  | *0.015* | *0.069* |
|  | No | 0.664 [0.200; 2.206] | 0.504 |  |
|  | Do Not Know | 0.286 [0.122; 0.699] | 0.004 |  |
|  | Yes | Reference^b^ |  |  |
|  | *Teacher* |  | *0.026* | *0.097* |
|  | 0% | 13.125 [1.990; 86.554] | 0.007 |  |
|  | 5-25% | 4.800 [0.700; 32.903] | 0.110 |  |
|  | 30-50% | 5.625 [0.688; 46.019] | 0.107 |  |
|  | 55-75% | 3.500 [0.372; 32.971] | 0.274 |  |
|  | 80-100% | Reference^b^ |  |  |
|  | *Work Sector* |  | *0.005* | *0.086* |
|  | Public | 4.700 [1.346; 16.411] | 0.015 |  |
|  | Private | 7.133 [2.195; 23.187] | 0.001 |  |
|  | Academic | Reference^b^ |  |  |
| Formal Training in Search Strategies |  |  |  |  |
|  | *Age* |  | *0.007* | *0.089* |
|  | 20-29 | 5.262 [1.941; 14.260] | 0.001 |  |
|  | 30-39 | 3.577 [1.521; 8.410] | 0.003 |  |
|  | 40-49 | 3.462 [1.168; 10.263] | 0.025 |  |
|  | ≥ 50 | Reference^b^ |  |  |
|  | *Years of License* |  | *0.012* | *0.081* |
|  | < 5 | 3.832 [1.276; 11.508] | 0.017 |  |
|  | 5-10 | 3.283 [1.475; 7.307] | 0.004 |  |
|  | 11-15 | 1.800 [0.806; 4.022] | 0.152 |  |
|  | > 15 | Reference^b^ |  |  |
|  | *Pursue a Higher*  *Academic Degree* |  | *0.016* | *0.059* |
|  | No | 0.447 [0.181; 1.108] | 0.082 |  |
|  | Do Not Know | 0.370 [0.177; 0.772] | 0.008 |  |
|  | Yes | Reference^b^ |  |  |
| Knowledge of Online Databases |  |  |  |  |
|  | *Pursue a Higher*  *Academic Degree* |  | *0.004* | *0.099* |
|  | No | 0.393 [0.124; 1.242] | 0.112 |  |
|  | Do Not Know | 0.223 [0.092; 0.541] | 0.001 |  |
|  | Yes | Reference^b^ |  |  |
| Formal Training in Critical Appraisal |  |  |  |  |
|  | *Age* |  | *0.043* | *0.062* |
|  | 20-29 | 4.230 [1.447; 12.370] | 0.008 |  |
|  | 30-39 | 2.547 [1.060; 6.119] | 0.037 |  |
|  | 40-49 | 3.322 [0.999; 11.045] | 0.050 |  |
|  | ≥ 50 | Reference^b^ |  |  |
| Confident in Appraisal Skills |  |  |  |  |
|  | *Clinical Instructor* |  | *0.039* | *0.038* |
|  | Yes | 2.301 [1.042; 5.085] |  |  |
|  | No | Reference^b^ |  |  |
| Confident in Search Skills |  |  |  |  |
|  | *Years of License* |  | *0.035* | *0.099* |
|  | < 5 | 1.926 [0.205; 18.103] | 0.556 |  |
|  | 5-10 | 0.254 [0.078; 0.824] | 0.023 |  |
|  | 11-15 | 0.267 [0.078; 0.916] | 0.036 |  |
|  | > 15 | Reference^b^ |  |  |
|  | *Clinical Instructor* |  | *0.008* | *0.070* |
|  | Yes | 3.272 [1.370; 7.815] |  |  |
|  | No | Reference^b^ |  |  |

^a^ Nagelkerke R^2^; ^b^ In logistic regression, one level of the independent variable serve as reference against which the odds of the other levels occurring are determined.

Table 8 – Association Between PTs’ Characteristics and Their Understanding of Scientific Terms.

| **Term (Do Not Understand)** | **Factor - Level** | **Odds Ratio (95% CI)** | ***P*** | ***R*^2 a^** |
| --- | --- | --- | --- | --- |
| Publication Bias |  |  |  |  |
|  | *Clinical Instructor* |  | *0.024* | *0.068* |
|  | Yes | 0.262 [0.082; 0.836] |  |  |
|  | No | Reference^b^ |  |  |
|  | *Work Mode* |  | *0.020* | *0.058* |
|  | Own Account | 3.339 [1.205; 9.252] |  |  |
|  | Some Else Account | Reference^b^ |  |  |

^a^ Nagelkerke R^2^; ^b^ In logistic regression, one level of the independent variable serve as reference against which the odds of the other levels occurring are determined.

Table 9 – Association Between PTs’ Characteristics and Their Perceived Barriers.

| **Barriers**  **(Present)** | **Factor - Level** | **Odds Ratio (95% CI)** | ***P*** | ***R*^2 a^** |
| --- | --- | --- | --- | --- |
| Insufficient Time |  |  |  |  |
|  | *Sex* |  | *0.016* | *0.042* |
|  | Male | 0.433 [0.220; 0.855] |  |  |
|  | Female | Reference^b^ |  |  |
|  | *Years of License* |  | *0.037* | *0.063* |
|  | < 5 | 3.106 [1.031; 9.356] | 0.044 |  |
|  | 5-10 | 2.941 [1.297; 6.668] | 0.010 |  |
|  | 11-15 | 2.000 [0.863; 4.634] | 0.106 |  |
|  | > 15 | Reference^b^ |  |  |
|  | *Clinical Instructor* |  | *0.029* | *0.036* |
|  | Yes | 0.484 [0.252; 0.930] |  |  |
|  | No | Reference^b^ |  |  |
| Lack of Information Resources |  |  |  |  |
|  | *Work Mode* |  | *0.022* | *0.039* |
|  | Own Account | 2.365 [1.133; 4.936] |  |  |
|  | Someone Else Account | Reference^b^ |  |  |
| Lack of Research Skills |  |  |  |  |
|  | *Work Mode* |  | *0.022* | *0.039* |
|  | Own Account | 2.365 [1.133; 4.936] |  |  |
|  | Someone Else Account | Reference^b^ |  |  |
| Inability to Apply Research to Individual Patients |  |  |  |  |
|  | *PTs in the Facility* |  | *0.008* | *0.105* |
|  | 0 | 3.491 [1.096; 11.124] | 0.034 |  |
|  | 1-5 | 4.945 [1.654; 14.790] | 0.004 |  |
|  | 6-10 | 2.200 [0.622; 7.787] | 0.221 |  |
|  | 11-15 | 12.800 [2.545; 64.372] | 0.002 |  |
|  | > 15 | Reference^b^ |  |  |
|  | *Work Sector* |  | *0.042* | *0.045* |
|  | Public | 0.271 [0.076; 0.986] | 0.044 |  |
|  | Private | 0.538 [0.168; 1.813] | 0.318 |  |
|  | Academic | Reference^b^ |  |  |
| Lack of Collegial Support |  |  |  |  |
|  | *Patients Day* |  | *0.038* | *0.067* |
|  | 1-5 | 0.280 [0.083; 0.950] | 0.041 |  |
|  | 6-10 | 0.686 [0.307; 1.532] | 0.358 |  |
|  | 11-15 | 1.426 [0.620; 3.280] | 0.404 |  |
|  | > 15 | Reference^b^ |  |  |
|  | *Work Mode* |  | *0.022* | *0.043* |
|  | Own Account | 0.376 [0.163; 0.867] |  |  |
|  | Someone Else Account | Reference^b^ |  |  |

^a^ Nagelkerke R^2^; ^b^ In logistic regression, one level of the independent variable serve as reference against which the odds of the other levels occurring are determined.
