# Supplementary material for: Measuring evidence-based practice in physical therapy: a mix-methods study
Source: PeerJ. 2022 Jan 4;10:e12666. doi: 10.7717/peerj.12666 (PMC8740513; doi:10.7717/peerj.12666)
Supplement: Supplemental Information 6 — Instructions for Submissions a–Relative risk; b–Absolute risk; c–Systematic review; d–Odds ratio; e–Meta-analysis; f–Confidence interval; g–Heterogeneity; h–Publication bias; α–Insufficient time; β–Lack of information resources; γ–Lack of research skills; δ–Poor ability to critically appraise the literature; ε–Lack of generalizability of the literature findings to my patient population; θ–Inability to apply research findings to individual patients with unique characteristics; σ–Lack of understanding of statistical analysis; ψ–Lack of collective support among my colleagues in my facility; ω–Lack of interest; NA–Not Applicable. [file peerj-10-12666-s006.docx]

Table 10 – EBP Questionnaire responses

| **Item** | **Response options (n; %)** | | | | | | | | | | | | |
| --- | --- | --- | --- | --- | --- | --- | --- | --- | --- | --- | --- | --- | --- |
|  | *Strongly Disagree* | | *Disagree* | | | | | *Agree* | | | | *Strongly Agree* | |
| 25 | 3 (1.6%) | | 1 (0.5%) | | | | | 90 (46.6%) | | | | 99 (51.3%) | |
| 26 | 2 (1.0%) | | 3 (1.6%) | | | | | 100 (51.8%) | | | | 88 (45.6%) | |
| 27 | 3 (1.6%) | | 25 (13.0%) | | | | | 111 (57.5%) | | | | 54 (28.0%) | |
| 28 | 17 (8.8%) | | 112 (58.0%) | | | | | 51 (26.4%) | | | | 13 (6.7%) | |
| 29 | 2 (1.0%) | | 8 (4.1%) | | | | | 125 (64.8%) | | | | 58 (30.1%) | |
| 30 | 2 (1.0%) | | 8 (4.1%) | | | | | 101 (52.3%) | | | | 82 (42.5%) | |
| 31 | 9 (4.7%) | | 85 (44.0%) | | | | | 86 (44.6%) | | | | 13 (6.7%) | |
| 32 | 38 (19.7%) | | 87 (45.1%) | | | | | 61 (31.6%) | | | | 7 (3.6%) | |
| 33 | 17 (8.8%) | | 107 (55.4%) | | | | | 63 (32.6%) | | | | 6 (3.1%) | |
| 34 | 1 (0.5%) | | 11 (5.7%) | | | | | 129 (68.8%) | | | | 59 (26.9%) | |
| 35 | 16 (8.3%) | | 99 (51.3%) | | | | | 66 (34.2%) | | | | 12 (6.2%) | |
| 40 | 2 (1.0%) | | 38 (19.7%) | | | | | 109 (56.5%) | | | | 44 (22.8%) | |
| 41 | 3 (1.6%) | | 25 (13.0%) | | | | | 124 (64.2%) | | | | 41 (21.2%) | |
| 44 | 3 (1.6%) | | 23 (11.9%) | | | | | 139 (72.0%) | | | | 28 (14.5%) | |
| 48 | 33 (17.1%) | | 56 (29.0%) | | | | | 82 (42.5%) | | | | 22 (11.4%) | |
| 49 | 9 (4.7%) | | 23 (11.9%) | | | | | 93 (48.2%) | | | | 68 (35.2%) | |
| 50 | 13 (6.7%) | | 47 (24.4%) | | | | | 84 (43.5%) | | | | 49 (25.4%) | |
| 51 | 3 (1.6%) | | 27 (14.0%) | | | | | 86 (44.6%) | | | | 77 (39.9%) | |
| 52 | 7 (3.6%) | | 37 (19.2%) | | | | | 92 (47.7%) | | | | 57 (29.5%) | |
| 53 | 1 (0.5%) | | 31 (16.1%) | | | | | 121 (62.7%) | | | | 40 (20.7%) | |
| 54 | 1 (0.5%) | | 28 (14.5%) | | | | | 124 (64.2%) | | | | 40 (20.7%) | |
|  | *≤1* | *2–5* | | | | *6–10* | | | *11–15* | | | | *≥16* |
| 36 | 35 (18.1%) | 87 (45.1%) | | | | 42 (21.8%) | | | 10 (5.2%) | | | | 19 (9.8%) |
| 37 | 41 (21.2%) | 94 (48.7%) | | | | 27 (14.0%) | | | 8 (4.1%) | | | | 23 (11.9%) |
| 38 | 54 (28.0%) | 68 (35.2%) | | | | 35 (18.1%) | | | 11 (5.7%) | | | | 25 (13.0%) |
|  | *Yes* | | | | *No* | | | | | *Do Not Know* | | | |
| 39 | 151 (78.2%) | | | | 24 (12.4%) | | | | | 18 (9.3%) | | | |
| 42 | 164 (85.0%) | | | | 29 (15.0%) | | | | | NA | | | |
| 43 | 161 (83.4%) | | | | 32 (16.6%) | | | | | NA | | | |
| 45 | 43 (22.3%) | | | | 150 (77.7%) | | | | | NA | | | |
| 46 | 134 (69.4%) | | | | 54 (28.0%) | | | | | 5 (2.6%) | | | |
| 47 | 175 (90.7%) | | | | 14 (7.3%) | | | | | 4 (2.1%) | | | |
|  | *Understand Completely* | | | | *Understand Somewhat* | | | | | *Do Not Understand* | | | |
| 55_a_ | 111 (57.5%) | | | | 72 (37.3%) | | | | | 10 (5.2%) | | | |
| 55_b_ | 114 (59.1%) | | | | 69 (35.8%) | | | | | 10 (5.2%) | | | |
| 55_c_ | 167 (86.5%) | | | | 24 (12.4%) | | | | | 2 (1.0%) | | | |
| 55_d_ | 56 (29.0%) | | | | 97 (50.3%) | | | | | 40 (20.7%) | | | |
| 55_e_ | 126 (65.3%) | | | | 58 (30.1%) | | | | | 9 (4.7%) | | | |
| 55_f_ | 129 (66.8%) | | | | 58 (30.1%) | | | | | 6 (3.1%) | | | |
| 55_g_ | 147 (76.2%) | | | | 39 (20.2%) | | | | | 7 (3.6%) | | | |
| 55_h_ | 110 (57.0%) | | | | 66 (34.2%) | | | | | 17 (8.8%) | | | |
| Total | 960 (62.2%) | | | | 483 (31.3%) | | | | | 101 (6.5%) | | | |
|  | *1^st^* | | | *2^nd^* | | | *3^rd^* | | | | Total | | |
| 56_α_ | 96 (68.6%) | | | 15 (10.7%) | | | 29 (20.7%) | | | | 140 (24.2%) | | |
| 56_β_ | 10 (22.7%) | | | 24 (54.5%) | | | 10 (22.7%) | | | | 44 (7.6%) | | |
| 56_γ_ | 14 (31.8%) | | | 15 (34.1%) | | | 15 (34.1%) | | | | 44 (7.6%) | | |
| 56_δ_ | 6 (21.4%) | | | 11 (39.3%) | | | 11 (39.3%) | | | | 28 (4.8%) | | |
| 56_ε_ | 25 (27.5%) | | | 38 (41.8%) | | | 28 (30.8%) | | | | 91 (15.7%) | | |
| 56_θ_ | 23 (22.3%) | | | 45 (43.7%) | | | 35 (34.0%) | | | | 103 (17.8%) | | |
| 56_σ_ | 8 (19.0%) | | | 17 (40.5%) | | | 17 (40.5%) | | | | 42 (7.3%) | | |
| 56_ψ_ | 10 (16.1%) | | | 20 (32.3%) | | | 32 (51.6%) | | | | 62 (10.7%) | | |
| 56_ω_ | 1 (4.0%) | | | 8 (32.0%) | | | 16 (64.0%) | | | | 25 (4.3%) | | |

a – Relative risk; b – Absolute risk; c – Systematic review; d – Odds ratio; e – Meta-analysis; f – Confidence interval; g – Heterogeneity; h – Publication bias; α – Insufficient time; β – Lack of information resources; γ – Lack of research skills; δ – Poor ability to critically appraise the literature; ε – Lack of generalizability of the literature findings to my patient population; θ – Inability to apply research findings to individual patients with unique characteristics; σ – Lack of understanding of statistical analysis; ψ – Lack of collective support among my colleagues in my facility; ω – Lack of interest; NA – Not Applicable.
