# Supplementary material for: Measuring evidence-based practice in physical therapy: a mix-methods study
Source: PeerJ. 2022 Jan 4;10:e12666. doi: 10.7717/peerj.12666 (PMC8740513; doi:10.7717/peerj.12666)
Supplement: Supplemental Information 7 [file peerj-10-12666-s007.docx]

Generally, the PTs shown positive beliefs, attitudes, knowledge, and behaviors about EBP. The majority of the PTs had similar EBP concept. Almost all of them included clinical experience, patient’s preferences and scientific evidence as the main actors (Quotation 1).

*Quotation 1: “EBP is a three-sided triangle. One, undoubtedly the base of the triangle, is the best available scientific evidence. Then we have, on the other side of the triangle, the clinician expertise. And we have the patient’s expectations on the third side of the triangle. When it’s possible to combine these three sides, in order to improve the treatment results or the intervention in that patient, then we do an EBP.” [FT_5]*

However, there were outliers cases. A PT beyond all the actors previously described, still found the necessity to add another one: ethics. For him/her, there is no EBP without ethics, being present and important at all times (Quotation 2). Furthermore, another PT, defined EBP as performing a practice in which the PT has to constantly register the clinical findings and makes his assessments according to international and national scales/tests (Quotation 3). The workplace may explain the reason for this different EBP perception. The PT worked in a continuing care unit, where scales/tests are used not only as a way of assessing, recording and monitoring the patients’ health status, but also as a way of communication between the several health professionals (the work in these units is mainly based on multidisciplinary health teams) (Quotation 4). In these units, scales/tests are also used as a basis for decision making to change the type of health service provided to a patient and to make a decision when to release a patient (Quotation 5). The other explanation may be related with the patients who attend to continuing care units. Where, patients are often very debilitated, with profound state losses, both physical and in consciousness. So, often, they are unable to participate actively in the treatment plan decision-making (Quotation 6).

*Quotation 2: “There’s no EBP without ethics. The PT’s ethics is a dimension that has to be involved in all moments of the EBP. And we continue to discuss very little about ethics in Portugal … and scientific evidence is one of the components of ethics. We often discuss scientific evidence, without discussing the basis, that is ethics. Whether it’s ethical or not ethical, proposing treatments that have no scientific evidence, and that’s the main basis of the discussion … EBP and ethics must always be associated.” [FT_4]*

*Quotation 3: “As we often say in our service: ‘What’s not detailed or registered isn’t done’. Based on the evidence, we use written validated things so that our intervention really has the needed validity.*

*(…)*

*Q: How is the current evidence regarding the majority of patients that you treat?*

*We ended up using in neurological cases, the Tinetti test and the Barthel index. Sometimes we generalize these scales to everyone. Of course, we still have strength tests, ROM tests…and the Borg scale, we also use it a lot …” [FT_6]*

*Quotation 4: “…who makes the evaluation is a team. (…) The team is constituted by doctors, psychologists, social workers, nurses and nutritionists and then, in the area of rehabilitation, there’s the PTs and occupational therapists, speech therapists and physiatrists. (…) … then at the next meeting, the individual intervention plan is defined, which is done in a team meeting, with the opinion of all areas …” [FT_6]*

*Quotation 5: “The individual plan is evaluated more or less in the middle of hospitalization, to see if it suit or not. Or if we have already achieved all the objectives outlined and we will propose others. Or, for any reason, if there’s a clinical situation worsening, the initial one is no longer applicable and we will have to outline other.” [FT_6]*

*Quotation 6: “Q: For you, what’s the patient’s role in EBP?*

*It depends on the patient’s state of consciousness. Because, often, disoriented patients arrive in the continuing care unit ... and don’t actively intervene in the intervention plan itself.” [FT_6]*

Still in the EBP concept, it is necessary to point out that there were PTs who balanced all the main EBP actors on equal importance levels and there were others who ranked their importance: evidence in 1^st^, patients’ preferences in 2^nd^ and clinical expertise in 3^rd^ (Quotation 7). A factor that could be involved in this ranking scheme is the PTs’ inexperience, since most of the younger PT reported it. In the clinical experience, it was stated that it only came with the years of work and the increased number of treated patients (Quotation 8). So, as younger and less experienced PTs still do not have many years of work, they cannot yet count on their clinical experience in the clinical decision-making (Quotation 9). However, this may not be the only factor responsible for this stratification. Some PTs who have higher academic degrees or who are currently increasing their degree, also made similar associations (however, in these cases, as in general they were more experienced, they placed clinical expertise in 2^nd^ and the patients’ preferences in 3^rd^) (Quotation 10).

*Quotation 7: “So I would say that I based my intervention plan on evidence (it’s my first contact to know what to do with that patient), then the patient values and characteristics comes afterwards, then my experience – that’s not that much (so I can only count on it to a certain extent) – comes in the end.” [FT_3]*

*Quotation 8: “Clinical experience only comes with time (…) So, what I mean is the number of patients, the clinical mileage, the increase in patients’ ‘kilometers’.” [FT_5]*

*Quotation 9: “I do this because I feel the need, both personal and professional, to ensure that everything I do has a justification and is based on something, which it’s not exclusively based on experience. Because, experience, I don’t have it yet.” [FT_2]*

*Quotation 10: “Our clinical decisions must be made taking into account the best evidence available, our clinical experience and, of course, also attending to the patient’ preferences and past treatments experiences. But always using this ranking. I. e., according to the evidence that exists, combine it with our clinical reasoning (in the light of what’s our clinical experience) and then adjust in a context that’s favorable for the patient.” [FT_7]*

So, the EBP concept can be influenced not only by personal but also by professional related factors. Besides the PT experience/inexperience and the increase of the academic degree factors, other elements were invoked by the PTs, namely: (1) the attendance to trainings/workshops/congresses; (2) the natural peer discussions; and, (3) reading and searching scientific papers. In fact, many PTs reported that, the EBP concept is not a fixed “element”, but it behaves almost like a caterpillar, i.e., it can transform from its foundation and metamorphoses with time (Quotations 11, 12, 13, 14, 15 and 16). The majority of the interviewed PTs had the foundations for EBP as part of their bachelor academic program, in either a specific lecture or part of the curricular internship (Quotations 17 and 18).

*Quotation 11: “… through discussions I been having, whether in Facebook professional groups, or in different PT congresses…then, it was mainly in the master’s degree that I learned the EBP importance …” [FT_1]*

*Quotation 12: “… I did my bachelor degree, completed a postgraduate degree and I am currently writing my master’s degree thesis. So I had many classes that focused on the investigation area, on EBP, on its importance and its limitations.” [FT_2]*

*Quotation 13: “I did a master degree and obviously in the master degree I gained respect for science and EBP. Then, I did my PhD degree and, in the PhD, the awareness of the value and importance of science and EBP became essential. It became a landmark. Nobody does a PhD without it. It’s really a crescent.” [FT_4]*

*Quotation 14: “Until the 3^rd^ year of the bachelor degree, we were thinking about recipes. That we were going to learn recipes to do anything and everything. We started to realize what we needed to know (at least what treatments can show results). Then, in the master degree the EBP concept was evidence, evidence, evidence. Only what was documented in the literature should be used. I think after the master degree and with the years of clinical experience, I started to incorporate other components, namely the patients’ preferences. They are, in fact, very important to achieve positive results (and as time goes on it ends up being increasingly important). So, at least regarding mental flexibility, things have changed over time.” [FT_8]*

*Quotation 15: “…when I finished the bachelor degree, I tried to justify everything, and everything I did would be through EBP. Now too, but maybe I try not to do it so hard. Because, many times, not everything we do can be justified. If we see that we have results, we will not stop doing it just because it’s not justified. I think that since the beginning, I have differentiated the concept, yes.” [FT_9]*

*Quotation 16: “I constantly do training, that forces me to read and increase my curiosity about some topics: ‘Oh, I’ve never heard of it, and I’m going to read’… in the past, I think PTs used to do the ‘5 holy grail’ [heat/ice, TENS, US, passive mobilization and massage]. Or for that situation you do this, for that you do that. I think nowadays, due to the fact that there are more studies, I think it has already changed a lot.” [FT_10]*

*Quotation 17: “The first contact was in the bachelor degree, in a lecture that we had about research in the 2^nd^ year of graduation (although, in the 1^st^ year we had already covered some clinical cases, where it was suggested that we search for scientific articles – but that was very difficult, because we didn’t have the base concepts). And then, in the 2^nd^ year, we approached more the EBP and the search in scientific databases.” [FT_2]*

*Quotation 18: “… in the curricular internship an item that was evaluated was whether our practice has, in fact, evidence based…at the internship level they, (I can’t say, obliged), but basically they encouraged us to do an EBP.*

*Q: But didn’t you had specific a lecture about EBP?*

*No, nothing. I don’t know if it exists at the moment, but there was none at my time. It was during normal academic lectures, that we were taught that. But at a general level. Not in a particular lecture.” [FT_9]*

Although some PTs balanced the main actors level of importance and others ranked their importance, for all, evidence has a very important role in EBP (Quotation 19). Evidence helps them to: (1) make more informed decisions; (2) justify their decision making; and, (3) have more certainty in the diagnosis, prognosis and intervention plan (Quotations 20 and 21). The majority associated the evidence with the *best evidence available at the moment* (Quotation 22). For that, it was mentioned that it was necessary to: (1) gather information; and, (2) critically analyze the information collected (Quotation 23).

*Quotation 19: “… for me, the basis will always be science, i.e., what we are going to do must be based on evidence. (…) Physical therapy is a health profession in the scientific field. So, everything we do must have a basis, and scientific evidence must be, the basis of everything else. And our experience is achieved on the top of the scientific evidence. And the patients’ preferences are also on top of scientific evidence.” [FT_8]*

*Quotation 20: “…what science has to say about it, i.e., what’s expected, what’s effective, what’s the prognosis, what should be done, what shouldn’t be done, what are the mechanisms, and so on…” [FT_1]*

*Quotation 21: “… justify the diagnosis that we probably attribute to the person, justify the prognosis (that’s often what most worries the person). And we cannot justify a prognosis based on our experience, we have to base it on what’s written, on the evidence.” [FT_2]*

*Quotation 22“… but in the light of the best evidence at the time, help us to be more certain that what we are doing is the best possible intervention…” [FT_3]*

*Quotation 23: “… it’s not enough for a PT to read a study … a study doesn’t mean anything … it’s necessary to read systematic reviews, meta-analyzes and guidelines, and then see if there’s good evidence, if there’s bad evidence, if there’s a lot, if there’s little, and what it points to.” [FT_1]*

The most frequent strategies that PTs use to keep updated were similar with those pointed as responsible for the EBP concept transformation: (1) the attendance to trainings/workshops/congresses; (2) natural peer discussions (either personal or by social media); (3) increase academic degree; and, (4) read and search scientific papers (either physical or by electronic databases) (Quotations 24, 25, 26 and 27). For the interviewed PTs there is not an exclusive way to gather information. All strategies were considered valid and important (Quotation 28). Nevertheless, preferences and advantages in the different evidence actualization strategies were stated. The pointed advantages of peer discussion are to have a different opinion of a topic (can change the personal perspective) and/or have access to a more specialized professional that can bring new, better informed and summarized evidence (Quotations 29 and 30). Similar to peer discussion, is the attending to congresses advantages. The PTs stated that going to congresses is a good way to receive summarized evidence and to be in contact with a new/innovator/not well-known treatment. Furthermore, as congresses can show new interventions, the PTs could later attend to workshops as, besides of being another example of having evidence summarized, it could help to transform the theory into practice, showing the PTs how to perform an intervention (Quotation 31). Regarding the practice-related Facebook groups, it is stated that there are useful studies’ sharing and discussion (Quotation 32). Increasing the academic degree can have an important role to keep up to date, since in most of the individual lectures their opinions must be based in the evidence and their thesis developed with the most recent and high quality evidence (Quotation 33). Besides being a good way to keep up to date, increasing the academic degree has another important role in evidence, since it improves the search performance and the ability to critically analyze studies (Quotation 34). Nevertheless, regardless that these strategies give a better and faster overall idea of a topic or area, most affirm that it lead them to go to the source – read and search scientific studies (Quotations 35 and 36). Although some receive studies directly via email (by subscription to scientific journals), most of them read and search scientific studies in online practice-related databases, being paper form the least stated option. The online databases were searched ranging from 1 per week to 5 per week (Quotations 37 and 38). The most common studies types searched were: CPGs, systematic reviews, meta-analyses, RCTs and expert opinion papers. From those, the PTs preferred to start reading information through studies that have a higher level of evidence, such as CPGs, systematic reviews or meta-analyzes (Quotations 39 and 40). Another factor that could interfered in the study type choice, may rely on the specific area or topic that is been searched. In fact, most of them raised daily clinical practice or academic related factors as the main reasons to do the searches (Quotations 41, 42, 43 and 44).

*Quotation 24: “… I try to follow practice-related Facebook groups or some more face-to-face forums …” [FT_1]*

*Quotation 25: “I subscribe to several physical therapy journals. Also, I follow blogs of people that I think are influential and important for me (for example former teachers) … and then through studies that I search on specific topics. Also books on specific topics that I buy when I need them. (…) Therefore, almost daily I receive emails with scientific studies…” [FT_3]*

*Quotation 26: “… in the PhD its [EBP] value and importance becomes fundamental.” [FT_4]*

*Quotation 27: “I think that going to international congresses have helped a lot …” [FT_8]*

*Quotation 28: “… I don’t know if there’s a better way. All are important, of course with different levels of knowledge acquisition …” [FT_5]*

*Quotation 29: “For me, the best strategy is peer discussion. To see others’ perspectives, others’ opinions, to listen to experts in the area…” [FT_1]*

*Quotation 30: “… I work with other colleagues that have an EBP and I ask them if they have read an article that was interesting, or that they recommend.” [FT_2]*

*Quotation 31: “Congresses are essential for people to hear things that they never heard before and hadn’t read yet. (…) Pubmed alone, has more than 900,000 publications per year, and it has more than 9,000,000 citations of entries per year of health-related articles, so it’s impossible to read everything. So, congresses help. Then, workshops help implementation in practice. This year, I happened to be at a very interesting congress on the ankle where I saw a different approach of the ankle treatment. (…) It’s a matter of time before it starts to be worldwide implemented. I personally started to implement it in my practice, I did some workshops right there that gave me some practical base …” [FT_5]*

*Quotation 32: “Also, in Facebook groups with other PTs, sometimes studies are shared that are quite interesting in other areas, which may not be my area of intervention, and for that sharing, sometimes I end up reading other studies.” [FT_2]*

*Quotation 33: “For me, it was the academic degree increase. The master degree contributed a lot. During the master degree, I was always reading about the topics I mastered the most. Then, I went to PhD and ended up reading even more and understand a little bit more about the evidence. For me, the academic degree increase has been very important in this aspect. Because, at least, it never let me stop updating in relation to the topics and areas that I like the most, or in relation to the areas where I work the most.” [FT_8]*

*Quotation 34: “We talked about something very important, increasing the academic degrees. I have the experience with this. It’s a brutal quality leap. For the practice, it depends on the level that you’re taking, master or doctorate degree. (…) But from the quality point of view of search, analysis, and willingness to improve knowledge, any of these level are fundamental. It’s logical that the doctorate level, has a different demand from the master. But, any of them, are a good start.” [FT_5]*

*Quotation 35: “… for my master degree Pubmed became a close friend. I search on it a lot, not that much for my clinical practice, but for lectures works and my thesis.” [FT_1]*

*Quotation 36: “In congresses you learn. Of course, for implementing it in practice it’s not enough. The person gets an idea, takes the authors name, and then goes home to study them.” [FT_5]*

*Quotation 37: “In average, 1 per week.” [FT_4]*

*Quotation 38: “Besides the weekend (and sometimes as well), during the week I search every day.” [FT_7]*

*Quotation 39: “Q: What kind of studies do you read the most?*

*Systematic reviews. And then, some more experimental research articles ...*

*Q: Including, guidelines?*

*Guidelines too!*

*Q: But fundamentally systematic reviews?*

*Yes. With or without meta-analysis. Systematic reviews, that’s what I end up reading the most. And then, some expert opinion papers.*

*Q: But never the first line of reading, correct?*

*No, the first line of reading is, objectively, systematic reviews.” [FT_4]*

*Quotation 40: “Of course, the higher up the pyramid, the better. Naturally, systematic reviews of RCTs have the greater validity, and then descend until the case studies, or expert opinions.” [FT_7]*

*Quotation 41: “… when I have a new case in a not so well known area I do search …” [FT_1]*

*Quotation 42: “Usually, it’s more according to my academic needs. As a teacher. The studies that I end up reading have more to do with my academic part and with the topics that I’m teaching. But, obviously, they cover with useful and effective information for my clinical practice.” [FT_4]*

*Quotation 43: “I always try, especially if it’s a pathology, or a patient with specific characteristics, to do some research in Cochrane, Pubmed, or other databases. Understand in terms of clinical trials, RCTs, systematic reviews, guidelines, what exists within that area …” [FT_7]*

*Quotation 44: “I read at least one study per day. Sometimes, I read more … it depends on the new cases, pathology and necessity that I have at the time.*

*Q: So, you do your search related with your patients’ pathologies, right?*

*Majority, yes.*

*Q: Did you have interest to do the search by intervention?*

*Sometimes, I do. But it’s rare.” [FT_10]*

Despite PTs placed evidence as a key element in EBP, sometimes they cannot count on it. The reasons raised were: (1) they cannot reach the best evidence available; (2) the evidence is often inconclusive; (3) as the evidence is rapidly and constantly evolving, they can never be completely updated and informed; (4) it is difficult to apply in the daily clinical practice; (5) in the physical therapy area, it is difficult to find high quality studies; and, (6) there are still areas that are not fully explored scientifically (Quotations 45, 46, 47, 48 and 49).

*Quotation 45: “For example, in the geriatric area, where I work, sometimes the literature is very difficult to apply … (…) I think that we, unfortunately, are never very updated. Because there are always new studies coming out in a lot of journals. I think that, afterwards, it’s a very constant job, trying to know what’s new. And for that, I have some difficulty. I go on searching, depending on the pathologies that I’m following, but sometimes I can’t be as updated as desired. (…) Sometimes, we must also realize that, in addition to understand the studies’ limitations and their level of evidence, it’s even more important to understand that not all studies are possible to extrapolate. Although, for example, the person that we are treating may be similar to an intervention group that we saw in a study, we must understand that it’s not always possible to extrapolate. Because the person may have other co-morbidities that imply their clinical condition.” [FT_2]*

*Quotation 46: “... of course the evidence is constantly evolving, and what it’s valid today, isn’t quite like that tomorrow ... (…) And then the question of the physical therapy evidence strength. Has a lot to do with certain methodological parameters that are used to assess the quality of the evidence. For example, if the person applying the therapy is blind. All those parameters that we have on the scores that evaluate the evidence in physical therapy, aren’t always so easy to reach. Because, for example, a PT knowing what he/she is applying. Certain parameters aren’t easy to achieve, i. e., many of the scores levels that exist in the evidence end up not being the highest ones ... (…) So, in one hand, there are still few studies and, in the other hand, the studies’ scores aren’t always the highest.” [FT_3]*

*Quotation 47: “... it’s difficult to compare the results between studies in physical therapy ... because the evaluation methodologies in each study are very different, the intervention strategies are very different and then it becomes very difficult to evaluate the results properly. (...) We have difficulty in explaining and validating our strategies because they are always different and the ways of presenting the results are also different and then it becomes difficult to compare them.” [FT_4]*

*Quotation 48: “… because every time that we read a study, with rare exceptions, the systematic reviews conclusions are always the same: studies with weak quality, inconclusive, lack of coherence between studies.” [FT_5]*

*Quotation 49: “… it depends on the areas. There are areas that are extremely well studied and extremely well-founded. And there are others that are very gray areas.” [FT_9]*

So, in those cases, they have to rebalance the importance of the other two actors (clinical experience and patients’ preferences) to make their clinical decisions. Clinical experience may help PTs to know quicker what could result in that specific type of patient, to define which therapies they master the most and make more assertive diagnostics and prognostics (Quotations 50 and 51). In other hand, in the patients’ preferences, it was stated that it is important to listen to the patient as: (1) each patient is unique, so after gathering the information by evidence and clinical practice the intervention plan has to be adapted to the patient’ preferences and needs; (2) in case of doubt, the patient’ preferences could help to establish the interventions applied; and, (3) as the treatments are applied to the patients, the intervention plan final word is always theirs (Quotations 52 and 53).

*Quotation 50: “Throughout life, what’s intended is that, in continuous professional development, the PT continues to absorb science and practice science. And then compare the results of his practice with what was expected of science and realize that maybe it’s more competent in some strategies and get more results, and maybe less competent in other strategies so doesn’t get so many results. (...) Experience can make the diagnostic process much easier. Can be much more assertive in the diagnosis in physical therapy with more experience. As for the intervention, experience will give us more effectiveness in the intervention, better results in less time, but it must always be based on scientific evidence. Clinical experience will tell me: ‘I’m better in certain strategies and I’m not so good in other strategies’. And, therefore, I will use the strategies where I’m better.” [FT_4]*

*Quotation 51: “On the other hand, I think that with more years of experience it’s easier (and with a better degree of certainty) for PT to choose a more adequate intervention plan.” [FT_8]*

*Quotation 52: “The patient’s role is fundamental, because the patient has the final word.” [FT_3]*

*Quotation 53: The patient’s role is fundamental! Fundamental, in the way that my intervention will be directed to the patient's needs and problems. Therefore, I have to accord with the patient what he/she wants to see resolved. It’s not what I want to see resolved as a professional, but it’s what he/she wants to see resolved as a patient. Therefore, he/she has to be very clear in what he/she wants and I have to present the intervention strategy. (…) ‘I have ‘X’ time to solve the problem, what strategies can I apply in this time?’; ‘I have more time, I maybe use this type of strategy, I have less time, I use another one.’; ‘What do I want?’; ‘What can I do?’. Therefore, the patient is fundamental. It’s the fundamental part! Physical therapy has to center its care based on the patient. (…) Each patient is unique…” [FT_4]*

However, as exposed, in some cases one or more actors could fail (for example, evidence nonexistence or inconclusive, not enough clinical experience or patient with a poor level of consciousness). Therefore, the PTs never counted in each actor individually to make clinical decisions (Quotations 54, 55 and 56). Even in a mental ranking scheme, when possible, they tried to incorporate all actors to make a final clinical decision. However, not always shown to be a simple and easy task.

*Quotation 54: “… speaking of the less benign musculoskeletal conditions, the evidence clearly points, in the vast majority of them, to a very good prognosis and good spontaneous evolution. I think that there’s often an excess of treatment or excess of intervention in this level. (…) We have to be skeptical on our practice ... (…)* *we have to be skeptical to the point of realizing: ‘This got better, but it got better because I did it, or it got better because it was going to get better anyway?; Or is it better for something else?’ – I can’t prove it – therefore, we must always be informed by the evidence and be skeptical of the results we obtain.” [FT_1]*

*Quotation 55: “… especially when working with older population they have many beliefs that aren’t very actual …” [FT_3]*

*Quotation 56: “The scientific evidence exists in some studies, but with little scientific strength.” [FT_4]*

First, is the PT’s internal discussion, between evidence and clinical practice. The PTs stated that when a solid scientific prove about a topic (area, intervention or pathology) exists, the PT’s personal opinion should never overlap it (Quotation 57). Once again, ideally, the practice should be evidence guided. The main reasons stated were, in one hand, scientific papers have to respect rigorous quality methods to evaluate the outcomes and analyze data and, in the other hand, generally they have a larger number of enrolled patients in comparison to the daily clinical practice (Quotations 58 and 59). However, science still cannot answer and prove everything. The PT’s daily clinical experiences can show what intervention can improve the condition in a particular patient. If a PT has a lot of experience in treating specific cases (expert) and when applying a specific intervention plan he/she has good results, even if some of the therapies applied have a few studies or are poor quality, he/she feels confidence in maintaining that intervention plan (Quotation 60). Furthermore, the studies’ nature and conduction can be an issue in this relationship and poor evidence trust. Sometimes the research groups fail to analyze a treatment effectiveness in its wholeness (Quotation 61). A suggested way to improve it can be by basing/constructing/guiding the studies by the daily clinical practice doubts and make them easier to understand and apply (Quotations 62 and 63).

*Quotation 57: “If there’s a relatively solid proof of a certain thing, even if we have an opposite opinion, I think that our personal opinion should not superimposed science. What our opinion should do is, taking into account that information and taking into account what we know about our cases, adapt it to science and not the other way around (adapt science to our beliefs is almost making studies ‘cherry picking’ to try to prove what we think).” [FT_1]*

*Quotation 58: In the studies samples there exists millions or thousands of patients, so our practice turns out to be not ideal and highly biased.” [FT_1]*

*Quotation 59: “I often try to go with what’s written … I put more weight on the balance in the papers…we often believe that who do intensive studies is always a little more right than those who don’t.” [FT_10]*

*Quotation 60: “And I think that we can be neither ‘8 nor 80’. Not everything that we find in the literature is effective and not everything that’s not studied isn’t effective. (...) … imagine that I have 20 knee injury cases and I always establish the same exercise plan and, with that exercise plan, those 20 persons had good results. Although, perhaps, not all exercises are documented as useful, I can see that those exercises actually had an effect. We should try to make our practice evidence guided, but also not just rule ourselves by it. We always have to try to find alternatives.” [FT_9]*

*Quotation 61: “... I think that, often, the problem isn’t that the technique has results or not, but often it has to do with how we explain the technique. I'll give you an example…it might be easier. Let’s assume a massage technique. An issue of manual therapy always achieves very small effect sizes. There are always huge doubts about the effects of manual therapy. But a few years ago, the way it was explained was based on purely biomechanical reasoning ... let’s ‘loosen a contracture’. Today, the explanation is in light of the neurophysiological effects. If I consider that to obtain certain neurophysiological effects, massage is a good therapeutic resource, then I use it. Despite knowing that some manual therapy techniques have no evidence, if it aligns with the effects I want, i.e., if that mechanical stimulus I need influences the patient's nervous system, then I use it.” [FT_7]*

*Quotation 62: “I think the discussion between the academic and the working class is very important, because the studies must be guided by the daily clinical practice doubts and the daily clinical practice must be based by the recommendations of science.” [FT_1]*

*Quotation 63: “Sometimes more simplified studies are needed to help us in our daily practice ... more applicable relatively to treatments.” [FT_2]*

Secondly, and sometimes the most difficult, is to conjugate the patients’ preferences with either evidence or daily clinical practice (Quotation 64). For an intervention plan to work properly, the PT and the patient have to be in agreement (Quotation 65). No matter how well designed the intervention plan is, if the patient does not feel motivated to do it, it will most likely fail (Quotations 66 and 67). So, for the success of the intervention plan, a good relationship and proper communication between the PT and the patient is mandatory (Quotations 68 and 69). For this good communication, it is necessary for the PT to adapt its speech to the patient health literacy level (Quotation 70). Where, not only what will be the intervention plan designed should be explained, but also what the treatments’ goals are, remove pathology related myths, and educate the patient to manage its health-related issues independently (Quotation 71). When there is a communication failure between the PT and the patient, the most common inductee stated was the PT since he/she, more experienced in the area and aware of the best evidence, has the responsibility to clarify the patient, even when they have very strong and wrong beliefs (especially in elderly patients) (Quotations 72, 73, 74 and 75).

*Quotation 64: “Of course, sometimes combining the patient’s preferences with the best available evidence – if those preferences are subject to some beliefs, some prejudices or based on previous experiences – we have to choose a prior work on education, deconstruct some ideas that can be wrong, and try to show that, ‘ok’, the patient may prefer this, but we consider that in the light of what was our assessment the best solution is another one.” [FT_7]*

*Quotation 65: “Even we sometimes like and would like to apply certain things that we saw in a spectacular study and that we think will have a great result, many times people don’t want them, or often they are ‘standing back’. And we realize, even that we can apply it, it will not work in the same way …” [FT_3]*

*Quotation 66: “I always use this parallelism. From the moment when a person comes to us, this will be a co-joint work. We both have to ‘row’ in the same direction, and we must always be aligned because what interests me is to be – and sorry for the expression – Cristiano Ronaldo in applying a certain technique, the best in the world to do that, if I haven’t the patient with me. In other words, I believe that it’s necessary to reach a consensus here.” [FT_7]*

*Quotation 67: “If I have a patient who needs, for example, TENS but doesn’t like it, then I will not apply it, because I think I will make it worst by applying it. (…) … for example, I use electroacupuncture a lot…I think that it has interesting results. I have patients that, although I realize that they will feel better, but as they don’t like needles, the worst thing that I can apply to them. It increases their pain. So, in those patients, despite many studies show good results, I don’t use it.” [FT_10]*

*Quotation 68: “... there’s often the idea that who works on the basis of evidence don’t see the patient as a whole … or ceases to be concerned with the relationship with the patient, seeing only numbers. I don’t agree. Most of the evidence that exists shows that it’s super important to create a therapeutic relationship … an appropriate therapeutic alliance. Empathy – and the context – that’s created is super important. Work in this way isn’t going against what the evidence is, on the contrary.” [FT_7]*

*Quotation 69: “However, the choice and decision about treatment must be shared with the patients. So it makes perfect sense if in evidence there’s more than one option, that this is made known to the patient. And if the patient is properly informed, he/she will be able to have a shared view on the treatment.” [FT_8]*

*Quotation 70: “We have to adjust our communication to the knowledge that the patient brings. (…) It’s the PT's responsibility to adapt his/her language in order to make an assertive and positive communication with the patient.” [FT_4]*

*Quotation 71: “And, on one hand, inform and correct the information he/she brings. It’s obvious that, today, access to information is very easy for everyone. Currently, what any patient does with a problem, is going to the Internet and analyze everything that’s written or, at least read (doesn’t mean analyzing, but reading) what’s written about his/her problem or similar problems in which then they cannot find the difference. And therefore, it’s up to us, PTs, when we have this type of patients, to demystify the wrong information that they can bring, make an assertive communication, and inform and educate the patients about that situation, empower the patients with correct health information.” [FT_4]*

*Quotation 72: “It’s our role, as connoisseurs of science, to transmit what science says to them …” [FT_1]*

*Quotation 73: “…because there’s no patient adherence and this is a real barrier, especially when working with an older population that has many beliefs which, of course, aren’t always as current or aren’t always so ready for everything.” [FT_3]*

*Quotation 74: “It’s the PT’s problem, because he/she has to be an excellent health communicator.” [FT_4]*

*Quotation 75: “… then we have to see something, which is: ‘The patient doesn’t want to do it, why?’. This is the first step, to understand why: ‘Is it because he/she had a negative experience with that technique or approach?’; ‘Is it because he/she has a wrong idea about this approach?’. And then the work is done according to reason. Usually, in my first session this always happens: ‘This is the best I have to offer, it will happen this way, we will try to make gains here and there, using these strategies. Do you agree with this approach?’ I put everything ‘in the table’. If the patient does not agree, in case it’s possible to make some adaptations, without neglecting what’s the rational reasoning that led me to decide like that, perfect! If we see that there’s no agreement, of course, either I forward to a colleague who uses another technique that’s preferred by the patient, or the ideal scenario is that we can actually reach a consensus. But above all, start by understanding what’s the reason for the patient’s positioning. If it’s a previous negative experience, if it’s a wrong idea. First work on that part, or take care of that part, and then reach a therapeutic consensus.” [FT_7]*

Yet, PTs are not the only inductee. Patients can also be a barrier. Often, their interventions preferences are either placebo or harmful (Quotation 76). When placebo, what was mentioned was that the PTs, in the first place, tried to explain (in the light of science and clinical experience) why the intervention is considered placebo and why it is not the best alternative for the patient. But if, after the explanation, the patient insists on doing the placebo intervention, the PT could give in to the request by coming into an agreement with the patient, integrating more effective interventions in the intervention plan. Doing so, the PT can make the intervention plan more effective while consenting with the patient’s will, increasing the degree of satisfaction, confidence and motivation. As a note, it was recalled that placebo interventions have some kind of results, since if the patient believes that the intervention will make him/her better, he/she will in fact feel better. Nevertheless, over time, the PT would gradually leave the placebo interventions and focus more time on more effective interventions. It is important to do it because placebo interventions can, often, become addictive. Although, PTs only conducted this clinical reasoning if a very important condition was respected: the placebo intervention has to be not harmful (Quotation 77). If there is a harmful choice or attitude regarding an intervention, the first rational is to explain why it is harmful and try to demote the patient to do it (Quotation 78). If the patient insists, the PTs could have 3 options: (1) not do the harmful intervention (first, PTs have to be beneficent and, second, PTs have the right to refuse to apply an intervention if they believe that it goes against their principles or causes harm to the patient); (2) do the harmful intervention, if the patient signs the informed consent and assumes the responsibility; or, (3) do the intervention against the patient will (if the patient’s life is at risk) (Quotations 79, 80, 81, 82 and 83). Nevertheless, once again, the keywords in both situations is a good, simple, correct, informed and assertive communication between the PT and the patient.

*Quotation 76: “… patients’ preference ‘ok’, but in a way that’s not harmful and I think even if we have to do a treatment that will be considered a placebo no matter how much the patient demands it, we should be as frontal as possible and say that the effects aren’t that good…” [FT_1]*

*Quotation 77: “… what I normally do in these situations is to explain scientifically why this is a placebo but I say: ‘Ok, I’ve no problem doing that’ (as long as I think it won't do any harm) … but I use it as a strategy to get into more active therapies that I know they work. So, ‘yes, we do this, but we also do this and that’. And, generally, the patient ends up giving in. In the overall treatment, I always reinforce more active strategies and I try to empower the patient so that he/she, in face of what he/she thinks is good for him/her, also try other strategies and get him/her to have more control over his problem and not just depend on other placebo treatments that may, eventually, be addictive. (...) So, the placebo has results ... certainly it does ... but do that, do nothing, or do something else that we know that has no effect is often the same thing. If the patient believe that he/she will improve with that, he/she will actually improve…it’s important to go according to the patient’ expectations. So I think it’s a way of gaining the patient’s confidence and, from there, take him/her to what should be better ...” [FT_1]*

*Quotation 78: “But, for me, this is the most difficult! Dealing with the patient’s expectations, when his/her expectations are completely different. But our role as educators comes in, because the PT isn’t the ‘applicator of techniques’ and isn’t just a ‘caregiver’. The PT is also an educator.” [FT_5]*

*Quotation 79:”... for example, doctors have the Hippocrates oath that says that they mustn’t do anything that causes harm to patients ... and we, even if we don’t have it, I think we should go that way.” [FT_1]*

*Quotation 80:”* *First, and foremost, I try to explain. But if the patient wants to do it, what I have to think is whether it’s or not contraindicated. If contraindicated, I don’t do it and explain why I don’t do it. I have the right not to do it.” [FT_3]*

*Quotation 81: “We have to be beneficent, not maleficent, and fair. If the technique that the patient wants to do and believes in, will not be harmful to him/her ... I could allow the use of the placebo, and then move on to other strategies. Of course, without a doubt. I explain and inform the patient, but if he/she insists on doing that technique, he/she has to sign an informed consent. If I think it’s not maleficent, if I think it can have some result (even if it’s psychological), if I explain what it’s all about, then I would do it if he insists on it. If maleficent, no, I don’t do it.” [FT_4]*

*Quotation 82:”* *It’s logical that afterwards there are limits to everything. Patients’ beliefs. Certain religions – I don’t even know very well – but there are religions that don’t allow blood transfusions. This is a patient’s belief. It’s logical, if the patient has a life-threatening problem, a brutal hemorrhage, if he doesn’t take the blood transfusion he can die. The doctor has to make a decision ... even if the patient is unconscious. And, sometimes, it goes against the patient’s expectations.” [FT_5]*

*Quotation 83:”If I think it’s not beneficial, I explain, show and prove why it’s not beneficial or advised in that situation and, usually, I don’t do it. If the patient insists, he/she has to sign an informed consent.* *(...) if the patient insists a lot, as long as he/she assume responsibility, I do it ...” [FT_10]*

Despite there are some obstacles to perform an EBP, most of the PTs stated that they perform it (or at least try) in their daily practice (Quotation 84). Generally, they stated that they have the resources and the work environment needed to practice an EBP (Quotation 85). However, they also affirmed that there were some details that could be improved and even pointed some barriers that made it difficult to implement EBP at work. One of the most common was the self-evaluation and assuming the blame for not having enough neither knowledge, informatics skills nor actively produce scientific studies (Quotations 86, 87 and 88). Another repeated statement is working under a medical prescription, since it does not allow them to apply the idealized and more evidence-based interventions (Quotation 89). Then, another factor was frequently stated, the wrong or even lack of resources (to perform the interventions or to be up to date with the evidence) (Quotations 90 and 91). The resources problem can be explained by either lack of money to buy them, or can be a result of the PTs’ lack of knowledge (if the PTs do not update regularly they do not know what new treatment strategies exist or what new equipment is on the market to complement their workplaces) (Quotations 92 and 93). The typology of the workplace can also influence the implementation of EBP since, in some of them, there is too much bureaucracy associated and, often, time is guided to fill documentation and not so much to the patients’ treatments (Quotation 94). Lack of time can also be an important barrier as, among others, it can limit the PTs’ updating ability (Quotation 95). Something that was related to the lack of time, was the high number of patients treated daily. As the PTs have a high number of patients to treat daily, there is not much time left for each patient, thus makes it difficult to implement the idealized intervention plan (Quotation 96). Another factor related to patients is that they often go with very restricted ideas or come with bad habits from other health care units (without an EBP), making it an obstacle to implement evidence-based treatments (especially more active treatments) (Quotation 97).

*Quotation 84: “Q: Do you consider that you do an EBP in your professional context?*

*I try to do it whenever possible. I think that yes, I do, but it’s still not ideal. But it’s the best it can be right now.” [FT_1]*

*Quotation 85: “We have an open space that’s perfect to do exercises with most of the materials needed for exercising. We have cycle ergonomists, we have treadmills, we have good stretches. So I’m thinking in terms of treatment techniques, but we also have access to the internet to read things … (...) there’s no one to say: ‘You can’t go this way you have to go that way’. So, we are autonomous in our decisions. Most of the time, at least in musculoskeletal area, I get to do most of the treatments I idealized.”[FT_8]*

*Quotation 86: “First of all, more knowledge on my behalf…everything globally, from evaluation, prognosis, interventions ... I think I still have a lot to learn.” [FT_1]*

*Quotation 87: “I will assume my computer ignorance. Just for using Skype, I said: ‘Oh my God! I never spoke on Skype!’(…) Twenty years ago, informatics weren’t as evolved as nowadays. We didn’t use the computer like now. And I have some difficulty in reaching some fields ...” [FT_6]*

*Quotation 88: “... if we see the published studies the PTs, and I assume my own blame, aren’t as motivated as the nurses in making publications ... in doing studies ... it doesn’t exist.” [FT_10]*

*Quotation 89: “... I just can’t do an EBP when I’m ‘fixed’ to a medical prescription. (...) ... I have already worked in a clinic with a medical prescription context, in which there was no flexibility on the part of the doctor to manage prescriptions together ... and then I was unable to manage the treatments properly.” [FT_2]*

*Quotation 90: “Almost all. Regarding material, I think that sometimes I could have more of this or that – I even think that things are getting in the way – but overall I can’t complain much. (...) Some weights and resistance bands are missing, which sometimes I feel that if I had them it would be a bit more helpful.” [FT_1]*

*Quotation 91: “I don't have the right resources. It’s almost like homework. In other words, all the studies I search are done at home. External to my working hours, on my personal computer.” [FT_2]*

*Quotation 92: “... I wouldn’t say that the conditions are absolutely ideal, but I wouldn’t say that there’s a lack of conditions either. So the basic things exist, some need renovation, others could exist, but there’s no money for everything.” [FT_3]*

*Quotation 93: “Q: So, lack of money it’s not the reason why resources do not appear. Maybe it’s because you never thought or suggested buy new products or materials. Is it?*

*I think so. If we show our institution, where I work, that it’s really important, they buy it.” [FT_6]*

*Quotation 94: “Because when we have imposed rules that don’t let us ... let me see if I can explain ... the continuous care unit worked a lot with bureaucracy, bureaucracy, bureaucracy. The important thing was to have the papers in order, and not the patients’ wellbeing, literally. (…) Because, firstly, it was the papers and, secondly, it was the patients.” [FT_10]*

*Quotation 95: “... I don’t really know why I found these three databases, but I know that I read it somewhere and pointed them out, but I confess ... I haven’t explored them yet due to lack of time.” [FT_6]*

*Quotation 96: “... I was responsible for a nursing home with 52 elderly persons plus a continuous care unit with 24 patients. It was too many patients for only one person. Especially at the nursing home…because in the nursing home there’s a lot that could be done. And I wasn’t able to do everything I wanted, due to lack of time.” [FT_10]*

*Quotation 97: “Because patients often come with a slightly restricted mentality, even when they come from other institutions where it’s heat, massage, and other elements and ‘you’re walking out from here’. And if we want to do something a little bit different, we can have a little bit of difficulties. But I think it’s more in the first phase. After people get to know us, seeing that we actually have results, then they change their position a little bit. (...)*

*Q: So it’s more related to … for example, patients are more used to passive treatments and you try to apply more active therapies ...*

*Exactly, that's right. And until we definitely do it, it’s a little bit difficult. But once we can change it, they can see that it has actually worked, it’s much easier for us, I think.” [FT_9]*

In addition to these personal daily clinical practice related barriers, PTs also pointed out other barriers responsible for a non-EBP in Portugal. Although they think that it is moving in a good direction, all agreed that EBP was still not widely practiced in Portugal (Quotation 98). The 5 main barriers themes responsible for this were: Personal PT factors; Physical therapy profession in Portugal; Workplace; Evidence; Physical Therapy national schools.

*Quotation 98: “Q: Do you think that in Portugal, Portuguese PTs do an EBP?*

*I don’t think so. For what I see and talk with some colleagues, I think that probably, colleagues who actually do an EBP are still a minority. However, I think that, in recent years (mainly because of some changes and maybe even through dissemination), I notice a greater concern with these issues. So, this makes me hopeful on this issue.” [FT_7]*

Within the Personal PT factors theme 3 sub-groups were found: Attitudes and believes regarding evidence; Laziness; and Age. In the attitudes and believes regarding evidence group, it was referred as barriers that: (1) the PTs are outdated or do not want to know what evidence shows; (2) the PTs do not give enough weight on evidence for their daily clinical decisions; (3) the PTs do not do continuous training and education; and, (4) almost as a consequence, most of the PTs still do not have the capacity to search and critically appraise studies (Quotations 99, 100 and 101). Since working according to EBP requires hard effort, most of the interviewed consider that in Portugal the PTs are lazy as: (1) “if one intervention results in one patient, it should result in other patients, and I do not have to improve my knowledge”; (2) it is easier to perform an intervention that doing a proper clinical rational; and, (3) there is still not a culture of evaluation and registration performed by the PTs (Quotations 102, 103 and 104). They also thought that all these exposed barriers are more evident in the oldest PTs as, for example, they have less informatics skills (making the access to evidence difficult), did not had the EBP basis, do not have the necessary abilities to search in electronic databases, do not do continuous training and education, and as the PTs age increases the importance of evidence in their daily clinical decisions may decrease (Quotation 105, 106, 107, 108, 109 and 110).

*Quotation 99: “I think most of the time they aren’t even aware or don’t want to know. So, they finish the bachelor's degree and from there they have ‘fully access’ to work according to what they know. Sometimes, it’s not so much formal training, but a person try to keep up to date with what’s there, and it’s something that doesn’t happen at all yet. At least in the vast majority of the cases I know. (…) The easy answer is: ‘But I do it this way and it works and as long as the patient feels good’… totally disrespect for science is the main reason. Because if a PT looks for how science works and perceives that it’s his/her ethical obligation to provide what’s properly proven to the patient and not exactly what I think is best. When the PT realizes this, he/she starts to come up with another clinical rational. I believe that the main reason is this, that a lot of people doesn’t realize it and ends up saying: ‘Oh no, but this has results and it’s good … they taught me this and the person feels much better and therefore I will do this’ ...” [FT_1]*

*Quotation 100: “So, I think there are many PTs who have a hard time getting information. I think there are many PTs who don’t really have a scientific literacy. I. e., it’s difficult to read studies, it’s difficult to choose between good studies and less good studies, they fail to make an appraisal of what they read, and therefore, they also say that there’s evidence for everything …” [FT_8]*

*Quotation 101: “My other colleagues just don’t do training, they don’t read, they don’t want to know, they still think that heat in an ankle sprain is the best thing in the world ...” [FT_10]*

*Quotation 102: “…on the other hand it’s a matter of facilitism… ‘What’s it to do? TENS, US and heat?… ok, it’s fine for me’. A person doesn’t even have to think much, doesn’t have to do a clinical rational… the diagnosis and intervention are already done, so just apply and get to the end of the day and earn the same. (…) …there are many PTs who aren’t at all concerned and do it in the easiest way (or seems best to them), not taking evidence into account. Because, in fact, working based on evidence is a hard task, it’s necessary for a person to search, keep up to date, make an evaluation and have a clinical reasoning.” [FT_1]*

*Quotation 103: “... there’s often the PTs’ laziness. It’s easier to have a ‘recipe’ for everything and always apply the same way.” [FT_7]*

*Quotation 104: “On the other hand, in physical therapy it’s very easy to have results, or at least patients say they are better. And since there’s neither a culture of evaluation and reassessment, the PTs end up being happy with their own practice. Then, the patients get better, but maybe it wasn’t the direct result of the PT treatments ...” [FT_8]*

*Quotation 105: “One of them is a lot of misinformation and an inability to search in databases. This is what I see with my older colleagues. They don’t know how to search, they don’t know how to build a search phrase, they can’t even create a search base. For example, in Pubmed 500,000 articles appear that have nothing to do with what the person is looking for. They don’t even know how to close a search expression. Then, in older colleagues, what I feel, is that they have already tried the therapy ‘a, b, c and d’, and they know that, in cases of lower back pain it always goes well with that therapy and they can’t extrapolate a little bit and see what's new, or what might be better ...” [FT_2]*

*Quotation 106: “... I can tell you that I have colleagues who graduated like me in 1987 and I never saw them in a congress. I assure you that I have been to many congresses, especially to the physical therapy national congresses I went to all ...” [FT_5]*

*Quotation 107: “… now there exists all these electronic databases and when I was studying, we still didn’t have that much.” [FT_6]*

*Quotation 108: “... I have to say that in my age group they aren’t so concerned with training ...” [FT_7]*

*Quotation 109: “In older PTs, there’s not exactly an appreciation of evidence and training and, therefore, people end up being demotivated. As they never saw the training being valued, they never update. It’s not mandatory to do training and I think that makes anyone – who has other important areas in life – ending up disconnecting a little bit from the profession. Because, at least in the public sector, people continue to work regardless of their training or education level.” [FT_8]*

*Quotation 110: “I think that 12 years ago, when I finished my bachelor degree, in schools EBP was not discussed a lot.” [FT_10]*

Regarding the Physical Therapy profession in Portugal theme, one of the most stated barrier was that physical therapy in the country rules itself by “vogue” interventions and by alternative therapies (Quotations 111, 112 and 113). This can lead to profession disrespect and discredit, giving a bad image to society and to other health professionals, and even lead to usurpation of work-related functions (Quotations 114, 115, 116 and 117). Another important factor is the individual profession characteristic, since it focuses mainly on providing QOL and not quantity of life (being quantity of life more valued in the current national health system) (Quotation 118). Moreover, in the national health system, the PTs in the primary health care are still scarce (Quotation 119). With this image and importance toward society and the health system, along with the recent profession age in the country, it can result in one of the main barriers in the profession: the PTs’ salaries (Quotation 120). PTs’ salaries are low, which often leads PTs to work in multiple places at the same time (Quotation 121). As they have to work in multiple places at the same time, they have little free time for other tasks, namely do, search and read scientific literature (Quotation 122). Another problem resulting from this lack of time, but also related to low salaries, is the poor trainings/workshops/congresses attendance. In the PTs’ opinion, in addition to their limited time for trainings/workshops/congresses, those that exist are scarce and expensive for the national panorama (Quotations 123, 124 and 125).

*Quotation 111: “... PTs follow other professions, namely osteopathy, chinese traditional medicine, etc., which have very little evidence and which aren’t even our profession, and therefore don’t even make sense.” [FT_1]*

*Quotation 112: “... I believe that – perhaps it’s not just our profession – but it works a lot by the vogue interventions. In other words, there was a time when manual therapy took on a great preponderance (almost all training courses were in manual therapy). At this moment, we are turning a little more towards exercises. Therefore, I think it also has to do with the development and the changes that are happening in the profession.” [FT_7]*

*Quotation 113: “I think that PTs in Portugal like a lot alternative therapies, especially in private practice,* *and I think it destroys a little the real appreciation that our profession should have.” [FT_8]*

*Quotation 114: “... we are an emerging profession, with a few years in Portugal and as such, sometimes, we aren’t recognized by the peers. (…) But this has to do with the threats that we create for other professions. These professions are the ‘chiefs of the tribe’ and are the ‘sorcerers of the tribe’, who don’t want to be threatened ... they don’t want to have their position on risk. So we’ve been having a hard time. Look what happened, not so long ago, with the creation of professional bodies. I don’t know if you have an idea of this, but APFISIO is claiming the creation of an PTs profession body since 1999. How is it possible to be able to create the profession body 20 years later, when you had all the rights, all the requirements under the legislation? It’s weird, ‘right’?” [FT_5]*

*Quotation 115: “... but generally speaking in terms of society, the physical therapy idea is a little bit wrong. In other words, there’s still a lot of connection to manual therapy, to massages and I believe that a large part of the population – perhaps less informed – is a little mistaken of what we do and what differentiates us.” [FT_7]*

*Quotation 116: “I think we are still far from what we [PTs] and the profession could be and to the level that it could reach. I think that, for example, people still confuse us a lot with masseuses and that we only do massages. I. e., they think that this is just our work and not just a technique. (...) I think there are still many functions usurpations, particularly from the rehabilitation nurses.” [FT_9]*

*Quotation 117: “For example, at the nursing home it went very well, because there was a nurse who thought that physical therapy was an interesting area, and we worked as a team. At the continuous care unit, this was no longer the case. Physical therapy was completely set aside. And when you work with nurses, as nurses want to do anything and everything, it ends up not being able to give truly importance to the profession. (...) ... at the continuous care unit, for about 7 years, I was the one who did all the respiratory physical therapy. After a certain moment, nurses started to do it. ‘Why?’ Because 3 [nurses] did a specialization in rehabilitation, and then they thought they could do everything.” [FT_10]*

*Quotation 118: “... the PT is a movement specialist and works much more on QOL than quantity of life. And that also limits us in terms of revindication rights capacity. (…) But while the most evolved countries are already able to realize the importance of QOL for the country’s development, here in Portugal there’s still some difficulty in realizing this.” [FT_5]*

*Quotation 119: “... I think things are still very badly set up. Because primary health care at the moment isn’t providing answers. There are almost no PTs in primary health care ...” [FT_8]*

*Quotation 120: “I left school 15 years ago and at that time the PT was more recognized than he/she is today. For example, monetary we were going through a better phase. (...) I think that more and more people are looking for physical therapy although, monetarily, we sometimes have to go through some more difficult phases.” [FT_6]*

*Quotation 121: “… I would increase the PTs salary so they don’t have multiple jobs, running around, not having time for anything and subjecting themselves to precarious working conditions.” [FT_5]*

*Quotation 122: “But I also think that it’s very difficult to produce good studies in our daily practice. Because it’s very difficult to be able to comply with all parameters (treat patients and be able to give the treatments that patients are expecting to receive and are paying for), and still manage to find time to do a study.” [FT_9]*

*Quotation 123: “Because, for example, it can’t be required to PTs to do more training, to have an EBP, if they don’t even know very well what an EBP is. There are PTs who think that EBP is merely reading articles. And it’s not, as I explained earlier. If there exists training in this direction it would help.” [FT_5]*

*Quotation 124: “... but I think that here in Portugal there are very few ways for us to update. Indeed, there are some interesting trainings, but many of the trainings bring us nothing based on the evidence. And I think that here, in Portugal, there’s a lack of congresses/conferences where there’s sharing of really good information.” [FT_8]*

*Quotation 125: “... we also have to see that training isn’t that cheap. And there may be PTs that don’t have the financial capacity to participate on it ...” [FT_10]*

Then, in part related with the profession, is the Workplace. The workplace could be a barrier since, in some workplaces, the PTs have to treat many patients per hour, thus it becomes impossible to them to perform an EBP (Quotation 126). In addition, there is often a total lack of responsibility for the role of the PT in clinical decision-making (especially in workplaces with medical prescription). In some workplaces, the PT do not participate actively in the intervention plan design, they just have to apply it (Quotation 127).

*Quotation 126: “… in clinics subsidized by the national health system, the PT is required to treat 10 patients per hour. This will have to be regulated ... this can’t be happening. Also because there’s no way to do an EBP.” [FT_5]*

*Quotation 127: “As the system is assembled. The lack of the PT responsibility, in most clinics, regarding the expected results. The PT doesn’t have to decide which strategy to apply. In the physical medicine and rehabilitation clinics, the system devalues the PT intellectual capacity, his decision making and in his concern with health outcomes and scientific evidence. There’s, usually, a medical prescription and a strategy previously established. The PT doesn’t think, he/she only executes the technique and, therefore, doesn’t have to make decisions. By not having to make decisions in the intervention plan, he/she isn’t concerned with scientific evidence.” [FT_4]*

Lastly, there were the Evidence and Schools barriers. They were the least stated barriers. Despite this, PTs still pointed some difficulties in each one. Regarding the evidence barrier, in addition to the already explored studies’ nature and conduction, it was reported that, as the majority of the studies are written in English with particular expressions, a linguistic barrier could be encountered (Quotation 128). In the schools theme, the PTs think that more importance should be given to EBP, as they felt that this concept is still not sufficiently valued (Quotation 129). It was also pointed that schools should update more often their contents (Quotation 130).

*Quotation 128: “… nowadays if we want to read an article, it has to be in English ... maybe in 10, 7 are in English.” [FT_10]*

*Quotation 129: “Probably schools also don’t go in this direction, which will lead to the fact that PTs later don’t appreciate it. (...) I don’t know if in schools, we neglect the importance of EBP, the importance of a certain posture and attitude in situations. I don’t know if it’s neglected. But somehow the message doesn’t get there. On the other hand, there is the reality of the new generations. They are different generations, and perhaps the way to reach them is that it hasn’t yet been discovered. (...) Probably schools aren’t getting there ...” [FT_5]*

*Quotation 130: “I think that schools – speaking of my bachelor school – I think it’s quite outdated regarding the contents. Because a person should leave school with the best evidence available and this often doesn’t happen.” [FT_1]*

So, the PTs suggested some facilitators to overcome the identified barriers. The facilitators described were aggregated in 5 main themes: Workplace and Way of working; PT Attitudes and Beliefs; Physical Therapy in Portugal; Schools; and Professional Bodies.

In the first theme, Workplace and Way of working, something that PTs felt important to underline is that physical therapy is a scientific autonomous health profession, so there should be no restrictions on their participation in the patient’s intervention plan (Quotations 131 and 132). To further highlight this factor, it was also suggested that PTs evaluate and register more (Quotation 133). Undoubtedly, it is important to have oral conversations (either with patients or with other health professionals), but what is not registered tends to be forgotten. Additionally, the ability to perceive the patient’s evolution throughout the intervention plan may be lost (Quotation 134). Another important factor frequently stated was time. In this theme, time was related to having enough time to properly treat each patient (Quotation 135). With the greatest need to evaluate and register, along with the demand for more time per patient, another proposal for improvement has emerged: evaluation and treatments should not be carried out in the same session. They must have different days to be performed (Quotation 136). So, as it can be inferred, the health care units owners can play a very important role in the EBP implementation. Besides they have the moral duty to perform it in their health care units, they should: (1) encourage, motivate and even reward PTs who attend to trainings/workshops/congresses or academically update; and, (2) promote frequent patients’ cases discussion and studies’ sharing among colleagues (Quotations 137 and 138). Still in information sharing between colleagues, it was suggested that, ideally, teams should be constituted of older and younger PTs. It is important to have an age heterogeneity, since the older PTs could transmit their clinical experiences to the younger ones and, in return, the younger PTs could help the older ones with some technologies skills, namely search in electronic databases (Quotations 139 and 140).

*Quotation 131: “As an autonomous professional, without a doubt, it’s necessary to reinforce this information. It’s very important that people know it. Because we know, we fight for it, but then who contacts us doesn’t always know it and that’s important to communicate to the population. There’s a PT who is an independent professional, who can function as a first contact for your health, or as a second contact for your health and that professional will then stay in contact with the rest of the health team, namely the family doctor. In other words, autonomous but not isolated.” [FT_4]*

*Quotation 132: “The legislation allows PTs to make and develop their intervention without the need for a physiatrist’s prescription.” [FT_5]*

*Quotation 133: “Evaluate, evaluate, evaluate and register. (...) Never treat a patient without evaluating and recording everything you see. (...) So, I think that was the first thing I would do: implement mandatory registrations and mandatory assessment forms (more standardized it would maybe be easier).” [FT_10]*

*Quotation 134: “Not only an open discussion, but a written report should be mandatory, so that all patients who reach the PT have an entry report and an exit report. Not just verbal discussion. Verbal discussion is very important, so that the PT can discuss the clinical case with the physiatrist or with any other doctor. It’s essential. But more important than this discussion, is writing. Keep the written reports, what was the initial assessment, what was the moment of discharge from physical therapy ... what were the values ​​obtained at the beginning and what ​​were the values obtained at the end. Maintaining written information is essential, more important than oral [communication]. Because the oral [communication] is lost and doesn’t exist.” [FT_4]*

*Quotation 135: “... because the main resource for me, for an EBP, is time. It’s not impossible, but it’s much more difficult if a PT is treating many patients per hour. And having the opportunity, as I have, to treat one patient per hour, you can do a more EBP.” [FT_1]*

*Quotation 136: “Another thing that would be important is the physical therapy consultation. Physical therapy consultation should be instituted and mandatory. We must differentiate the consultation from the treatments. There should be an initial consultation, a reevaluation consultation, and a final consultation. It’s not possible to conceive a physical therapy patient consultation to be done at the same time as the treatment, and the treatment at the same time as the consultation, and then the discharge is made on the last day. It’s not always planned that well. The system should change and the physical therapy consultation should be mandatory. Once a week. On Monday the physical therapy consultation, then on Tuesday, Wednesday and Thursday the treatments. And every two weeks the intervention plan is reviewed, or every week, according to the PT objectives. Imagine that we have muscle strengthening to do in 3 weeks, we will apply, we will reevaluate, and in 3 weeks we will evaluate the results of muscle strengthening to see if we have progressed, if we haven’t progressed, or what we have done.” [FT_4]*

*Quotation 137: “I think it would make a difference, a motivation from bosses, so that there’s a constant update in terms of evidence. Whether by giving, for example, 10 min ... whatever ... during working hours for the PT to look for a study, or to clarify about any issue that arises from a patient, or some pathology. And in a certain way, sharing a scientific study between colleagues should be a constant thing. For example, in a team meeting there’s a study sharing that came out, on some topic that’s common to all.” [FT_2]*

*Quotation 138:” I think that, if PTs were obliged and valued for training and for updating their academic degree, it would be very good. (...) There should be more incentive for training ... (...) At first, I wasn’t exactly very well interpreted. I think I ended up winning a place by talking to my colleagues, saying why I did this why I didn't do it another way. And we started to have some important discussions, which I think helped a lot. Both for me to continue reading and keep updating, and for some of my colleagues that also want to know more. Right now, we have weekly meetings, where we talk about all patients, and the discussions end up helping a lot. Someone who has read something about a topic, or who knows more about one topic than others. It also helps the team a lot to keep us up to date. We also adopted a policy of when we have a new patient, we make a treatment plan with established goals and timing. In other words, we evaluate a patient who has this problem to be treated at this time, with the hope of trying to reach a certain goal. It help to better control the patients’ admissions and release. (…) I think team discussion of cases is important. What I would like to see in my service. Do case studies, monthly, or have some work scheduled on patients, on new techniques, on new thinking, on certain areas, to also share to the team. And I think that in the service if there’s more of this team dynamics, in the sense of doing the best job possible, this is encouraged, I think that people end up motivating themselves and also end up looking for more training and information.” [FT_8]*

*Quotation 139: “... when there’s some time between patients, what we sometimes try to do is, for example, searching in electronic databases that are a little bit simpler, such as Google Schoolar (I don’t like to use it so much, but for most people who don’t find it very easy to search in electronic databases, such as Pubmed, it turns out to be a little bit easier). For example, ‘about low back pain, this new article came out’. I have a 60-year-old colleague, who sometimes asks what’s new – because he/she has always treated patients in the same way – and sometimes I send her the study. If I think it’s a new article, and that I can thus influence his/her practice.*

*Q: And she accepts it well?*

*Yes, he/she accepts. But I think it’s because of his/her personality. Like another colleague, around 40 years old, who has some difficulty and sometimes asks for help to build search expressions, when he/she has to find something. And I explain how it’s done.” [FT_2]*

*Quotation 140: “I will tell you how this will improve even more. First, a tutored practice in the early years of the profession. I never reflected on how many years, I don’t know if it’s two, if it’s four, if it’s five, but a real tutored practice. (...) The ideal would be for a newly licensed PT to be with a more experienced PT and to work with him/her (but this isn’t always possible, I know very well … I know the reality of our country very well). But do as psychologists do. They have a tutor and every week, or every 15 days, or every month, young clinical psychologists go to their tutor to present and discuss cases, situations and others.” [FT_5]*

However, for this to work out it is necessary that PTs partly lose their pride, have an “open-mind” and ask for help (Quotations 141 and 142). This is precisely the first factor mentioned in the PTs’ Attitudes and Beliefs theme. Another important factor stated was that it is necessary for PTs to be aware that they are treating patients and to treat patients properly they have to do an EBP (Quotation 143). It is necessary to “return to the origins”, not to lose the physical therapy identity and what separates it from other health professions, and trying to avoid the use of alternative therapies or others with little scientific evidence (Quotations 144 and 145). In order to define which techniques should be applied (preferably those with good evidence), PTs will also have to be more proactive and do more studies (Quotation 146). With this, it could be closer to achieve the last factor described in this theme: specialization. PTs thought that there is an increasing need to create specializations in physical therapy, in order to provide the best treatment experience to their patients (Quotation 147).

*Quotation 141: “They have to ask for help! PTs have to understand this! There’s no problem! I’m not a temporomandibular expert, I never decided to invest time and money in temporomandibular. When two or three cases with temporomandibular appear, I send them to a specialist, a PT who only treats temporomandibular. PTs have to realize that we are taking care of patients! But anyway …” [FT_5]*

*Quotation 142: “Q: How can we prepare a country for an EBP?*

*I think that as long as the PTs don’t have an open mind to accept that we don’t know everything and that we have to be in constant formation, we will not be able to achieve this. (...) ... there’s one thing that I believe, that the students who are being trained at this moment, their way of EBP thinking is already different. And they already feel that we don’t know everything and that we need to study.” [FT_10]*

*Quotation 143: “You can only do an EBP! Any other type of practice isn’t acceptable. Any practice other than an EBP can diminish our image. Physical therapy isn’t quackery, it’s not art ... it’s science and, as such, only an EBP is acceptable. Any other type isn’t acceptable.” [FT_4]*

*Quotation 144: “... I think that working with an EBP is an excellent way of valuing it, compared to other health professions but also to the population. I think doing an EBP is important, and explaining and speaking the same language, and not talking so much about more esoteric things. (...) ... don’t mix, with the same professional being involved in several areas ... some with little evidence.” [FT_1]*

*Quotation 145: “PTs have to be the firsts to set an example, and positive results are undoubtedly the best way to value the profession ... (...) Therefore, what would be necessary is improving the physical therapy care. There are still many places where care doesn’t correspond to what physical therapy is.” [FT_3]*

*Quotation 146: “And then, if we see the studies, the PTs – and it’s pointed also to me – aren’t as motivated as the nurses, in making publications. (...) As long as it’s not the PTs that have this type of attitude, and show: ‘No! We are capable! We are going to publish, we are going to do studies.’, the things will not change.” [FT_10]*

*Quotation 147: “I think by specialization. For example, today I treated a lady who uses a dental device and the dentist who put the device on her found it strange that I say that headaches and neck pains may come from the device adjustments. So, there are many areas that are still unknown and that we can and should explore.” [FT_6]*

If all this PTs attitudes and beliefs regarding EBP could be implemented, the first factor on the Physical Therapy in Portugal theme could be achieved: improve the physical therapy image to the patients and other health professionals. This could be done in various ways, such as awareness actions for the society, advertising (in newspapers, radio, internet and television), working in multidisciplinary teams, but above all by promoting good work practices, including EBP (Quotations 148, 149, 150 and 151). Thus, it could result in a greater profession valorization and consequently better wages (Quotation 152).

*Quotation 148: “And even as some professions do, awareness actions on the street, television or radio advertising about the importance of physical therapy in general.” [FT_1]*

*Quotation 149: “I think that through a more intensive dissemination of the profession of our work and all of our areas to other health professionals and the general population. Because many people are unaware of the intervention areas that we have.” [FT_2]*

*Quotation 150: “At this point, in my opinion, an information campaign to the community about the value of physical therapy would be necessary. Advertising campaigns, clearly about: ‘What’s a PT?’; ‘What does he/she do?’; ‘Where can he/she be found?’; ‘How can you reach them?’. And really what’s it that can contribute to people’s health.” [FT_4]*

*Quotation 151: “How can we change? Well … showing quality. In other words, doing an EBP.” [FT_7]*

*Quotation 152: “If the PTs earned more money in their workplace, working 7 or 8 hours a day, starting at 8 a.m. and leaving at 4 p.m., maybe, after 4 until 6 or until 7, they could have time to do other things. (...) That’s why I was telling you to give and create better working conditions ... earning more money isn’t for the PTs to be richer ... it has nothing to do with it. It’s for them not to need to work at 2 or 3 workplaces to have a minimally decent standard living ... not to be in a standard living for survival.” [FT_5]*

The physical therapy Schools can also help to change the way of working, the PTs’ attitudes and beliefs and improve the physical therapy valorization in society. For this, it will be necessary to instill and prepare physical therapy students, from the beginning, for an EBP (Quotation 153). It will also be essential to normalize and regularize the schools’ academic content programs in the country (Quotation 154).

*Quotation 153: “It would most likely start at the base. I. e., I would be much more concerned with the way bachelors and masters degrees are taught. EBP should be promoted right from the beginning. Because it’s a matter of habit. If a person spend years doing the same thing, he/she gets used to do it that way. If you’re constantly encouraged to feed into knowledge, to enjoy searching, to enjoy seeing results and to understand why they happen, and to increase your knowledge, this has to be stimulated and taught right from the beginning. The habit has to be created, and of course, if we manage to change a little bit what the transmitted concepts are during the bachelor level, this will help a lot. Because we are going to train professionals with a different mind openness and a capacity for themselves, at some point, to feel the need to cultivate knowledge. That would be the first option for me.” [FT_7]*

*Quotation 154: “... another issue that I have already mentioned: the importance of schools in this attitude. I know that, at this moment, meetings are taking place between the physical therapy departments coordinators courses at national level in different schools to start finding a curriculum and a common practice in teaching physical therapy in different schools. I know they are doing that ... right now they are working on it. Hopefully, things will change.” [FT_5]*

Of course, the PTs Professional Bodies can facilitate all these factors and themes. First, although it is well forwarded, it must be fully operational (Quotations 155, 156 and 157). Without this, any other factor to be mentioned in this theme may fall apart. In addition to others that can be carried out, the PTs pointed out as important tasks to be performed by the professional bodies: (1) regulation of the professional activity (as for example, define profession functions and limit the number of patients treated per hour); (2) in order to maintain the physical therapy license valid, the PTs would have to show that they keep updating (additionally, an exam could also be performed every 2 years to evaluate PTs level of competence); and, (3) do more physical therapy trainings/workshops/congresses in Portugal (for example, to explain to older PTs how to search in electronic databases) (Quotations 158, 159, 160 and 161).

*Quotation 155: “... also having professional bodies doesn’t mean anything. The professional bodies must work, work well and on the right path.” [FT_1]*

*Quotation 156: “It would also be important to pressure national, political and health organizations to attribute more competencies to PTs. Because, at the moment, we have a highly qualified profession, but small competences are attributed at a professional level. It’s obvious that it’s necessary to have an assembled structure. We still have little political weight, but I believe now with the appearance of professional bodies, this can slightly reverse the weights of the balance and we can start being able to put a little more pressure on the institutions.” [FT_4]*

*Quotation 157: “... I now hope that with the PT professional bodies creation an important leap will take place.” [FT_5]*

*Quotation 158: “I think that above all, in addition to the trainings that usually come up with the technique ‘a, b, c and d’, I think that it could exist – maybe for PTs who have graduated along time ago, in which evidence wasn’t given so much importance – short trainings in order motivate people and teach them to search and to help modifying their practice. We also don’t always manage to change people’s beliefs, but at least we can change some knowledge that can help them. And I think that would make a difference in the clinical practice of PTs. Because I believe that younger PTs, who have been practicing for less years, it is easier, but for the older there’s a lack of someone or a training that gives them some kind of basis.” [FT_2]*

*Quotation 159: “The PTs professional bodies consider that it should be mandatory every ‘X’ years for PTs to show formal or informal training credits, verifying that the PT hasn’t been stagnant. And this is important to us, it’s important to the community and patients who really recognize our value.” [FT_4]*

*Quotation 160: “I think it should exist an entity regulating the practice. Because, if there’s nothing or nobody to regulate, everyone can do whatever he/she want. If there’s someone, superior to us, who says: ‘No! To maintain the quality of services, in order to be effective, at least as to be X time’. So, from then on, putting this rule to all of us, that people can also see the difference. (…) But the fact that we (as in other countries, for example, England), every 2 years have to take an exam or a test to validate our skills, as a PT. I think that if we, in order to be able to practice physical therapy, had to do that, we would more easily apply an EBP at work ...” [FT_9]*

*Quotation 161: “I hope that with the creation of the PT professional bodies we will have someone who says: ‘No! This function is in this professional category.’” [FT_10]*
